# Design of plasmonic Ag-TiO_2_/H_3_PW_12_O_40_ composite film with enhanced sunlight photocatalytic activity towards *o*-chlorophenol degradation

**DOI:** 10.1038/s41598-017-17221-4

**Published:** 2017-12-11

**Authors:** Nan Lu, Yaqi Wang, Shiqi Ning, Wenjing Zhao, Min Qian, Ying Ma, Jia Wang, Lingyun Fan, Jiunian Guan, Xing Yuan

**Affiliations:** 1 School of Environment, Northeast Normal University, Changchun, 130117 P.R. China; 2 College of Chemistry, Northeast Normal University, Changchun, 130117 P.R. China

## Abstract

A series of plasmonic Ag-TiO_2_/H_3_PW_12_O_40_ composite films were fabricated and immobilized by validated preparation technique. The chemical composition and phase, optical, SPR effect and pore-structure properties together with the morphology of as-prepared composite film are well-characterized. The multi-synergies of as-prepared composite films were gained by combined action of electron-capture action via H_3_PW_12_O_40_, visible-response induced by Ag, and Schottky-junction formed between TiO_2_-Ag. Under simulated sunlight, the maximal *K*_*app*_ of *o*-chlorophenol (o-CP) reached 0.0075 min^−1^ which was 3.95-fold larger than that of TiO_2_ film, while it was restrained obviously under acid condition. In the photocatalytic degradation process, ·OH and ·O_2_^−^ attacked preferentially *ortho* and *para* position of o-CP molecule, and accordingly the specific degradation pathways were speculated. The novel composite film exhibited an excellent applicability due to self-regeneration of H_3_PW_12_O_40_, well-protection of metal Ag° and favorable immobilization.

## Introduction

The increasing concerns on environment and energy crises have induced considerable researches on eliminating organic pollutants in a “green” method. Photocatalysis provides such a technique with the aid of sunlight, nevertheless, the practical application of photocatalyst, such as TiO_2_^1–3^, in wastewater treatment and air pollution control is limited by its inherent drawbacks i.e. poor visible light utilization efficiency and low quantum efficiency^4^. Consequently, different ways of modification have been employed in which deposition of polyoxometallates (POMs) has been proved to be an excellent way to enhance the photocatalytic activity of TiO_2_^5,6^. Moreover, POM can be self-generated via the redox cycle between POM and POM^−^, and its hexagonal cage structure can enable the reaction solution to flow through the molecules and contact with the active sites adequately. In our previous researches, a series of TiO_2_ based photocatalysts with different textural and morphological properties have been developed via depositing 12-tungstophosphoric acid (H_3_PW_12_O_40_) as a kind of POM with saturation Keggin^7–9^. The synergism between H_3_PW_12_O_40_ and TiO_2_ led to an efficient electron trap effect, consequently, the electrons can rapidly transfer from the conduction band of TiO_2_ to unoccupied W 5d orbit of POM, resulting in high degradation efficiency towards various organic pollutants including dyes (methyl orange, rhodamine B)^10,11^, endocrine disrupters (bisphenol A, phthalate esters)^12,13^. However, efficient use of visible-light is still an appealing challenge for POM-TiO_2_ composite catalysts.

Recently, a new class of TiO_2_ based photocatalysts have emerged via depositing plasmonic noble metal nanoparticles including silver (Ag), gold (Au), and platinum (Pt), to enhance the utilization of visible-light^14–18^. Compared with other photosensitizers such as organic dyes^19^, non-metal doping^20^ and narrow-gap semiconductors quantum dots^21^, the plasmonic noble metal nanoparticles possess incomparable advantages in term of the chemical stability, flexibility, and selectivity^22–25^. As a result of oscillation of the free conduction band electrons, surface plasmon resonance (SPR) effect enables free carrier to transport and harvest visible light without a requirement of favorable band alignment^26,27^. Moreover, the Schottky barrier formed between semiconductor and noble metal significantly benefits the separation of electron-hole pairs^28–30^. As an especially attractive surface plasmon metal, Ag has been widely used to modify TiO_2_ due to the properties of relatively low-cost, excellent conductivity, chemical stability, catalytic activity, and near-field enhancement^31–37^. Therefore, it is conceivable that introducing Ag into TiO_2_/H_3_PW_12_O_40_ may enhance the visible-light catalytic activity.

Moreover, current studies on photocatalysis are mainly based on powder-type photocatalysts, which severely hinders their practical applications due to the post-treatment problems in these systems such as separation, recovery and reuse. To overcome these disadvantage, much attention has been paid to explore immobilized TiO_2_-based film materials^38,39^. Among various immobilized methods, the sol-gel method has been utilized extensively^40,41^. However, the occurrence of reunion and the required high temperature treatment exert a significantly adverse influence on the morphology and circulation of the immobilized materials^42,43^. In our previous study, a validated sol-gel-hydrothermal route followed by a spin-coating method was established, which exhibited a high catalytic stability and remarkable recyclability^11,12^. The hydrolysis rate was efficiently controlled by adding glacial acetic acid to avoid the reunion. Meanwhile, programmed temperature hydrothermal method with relatively low temperature was designed to ensure the crystallization of TiO_2_ and Keggin structure of H_3_PW_12_O_40_ in the high-pressure reactor.

Thus, a recoverable plasmonic Ag loaded TiO_2_/H_3_PW_12_O_40_ composite film was designed in the current study and its photocatalytic activity was evaluated in terms of degrading *o*-chlorophenol (o-CP) under simulated sunlight. The morphology and structure of the composite film have been well-characterized; batch experiments were conducted to reveal the influence of Ag and H_3_PW_12_O_40_ loading amount, initial concentration and pH value of o-CP on the photocatalytic performance to the target reaction; the photocatalytic mechanism and possible degradation pathways of o-CP were discussed deeply; finally, the recyclability of the composite films was tested by three times’ o-CP degradation run.

## Methods

The titanium tetraisopropoxide (TTIP, 98%) was purchased from Sigma-Aldrich Corporation. H_3_PW_12_O_40_ (GR), isopropanol (AR), AgNO_3_ (AR), o-CP (AR) were purchased from China Pharmaceutical Group. Other chemicals were of reagent grade and applied without further purification. Double distilled water was utilized throughout the experimental procedures.

### Catalyst preparation

The preparation of TiO_2_/H_3_PW_12_O_40_ film is described in the previous studies^11,12^. On the basis, AgNO_3_ was dropped gradually into isopropanol solution during the stirring according to certain proportion with TTIP or H_3_PW_12_O_40_, and the remaining steps were the same as the previous reported method. The obtained hydrogel was spin-coated onto quartz substrate (50 mm × 15 mm × 1 mm), and the as-prepared composite film was denoted as Ag(x%)-TiO_2_/H_3_PW_12_O_40_(y%), in which x and y represented the loading amount of Ag (wt%: 0.5%, 1% and 2%) and H_3_PW_12_O_40_ (wt%: 5%, 10% and 15%), respectively. The unary TiO_2_ and binary Ag-TiO_2_, TiO_2_/H_3_PW_12_O_40_ films were also prepared with the above procedures.

### Catalyst characterization

The loading amounts of Ag and H_3_PW_12_O_40_ in the composite films were determined by a Leeman Prodigy Spec inductively coupled plasma atomic emission spectrometer. X-ray diffraction (XRD) patterns were obtained on a Rigaku D/max-3c X-ray diffractometer (Cu Kα radiation, λ = 0.15405 nm). UV-Vis diffuse reflectance spectra (UV-Vis/DRS) were recorded on a Cary 500 UV-Vis-NIR spectrophotometer. X-ray photoelectron spectroscopy (XPS) was performed on a VG-ADES 400 instrument with Mg Kα-ADES source at a residual gas pressure lower than 10^−8^ Pa. Raman scattering spectra were recorded on a Jobin-Yvon HR 800 instrument with an Ar^+^ laser source of 488 nm wavelength in a macroscopic configuration. Field-emission scanning electron micrographs (FESEM) were obtained using a JEOL 6340 F scanning electron microscope. Nitrogen porosimetry was measured by a Micromeritics ASAP 2020. Surface areas were calculated by Brunauer-Emmett-Teller (BET) equation. Pore size distributions were calculated by BJH model based on the nitrogen desorption isotherm (samples were degassed for 1 h under vacuum at 363 K, and then for 12 h at 473 K). Transmission electron microscope (TEM) micrographs, high resolution TEM (HRTEM), and selected area electron diffraction (SAED) micrographs were recorded by a JEM-2100F HRTEM at an accelerating voltage of 200 kV.

### Photocatalytic activity test

The photocatalytic degradation of o-CP was conducted in a home-made quartz photoreactor under the simulated sunlight provided by a PLS-SXE300 Xe lamp (300 W, Beijing Trustech Co. Ltd., China) placing *ca*. 15 cm above the reactor. The lamp was equipped with an IR cut filter to match the natural sunlight with the wavelength ranging from 320 to 780 nm and light intensity of 200 mW/cm^2^ measured by a radiometer (OPHIR, Newport, USA).

In the photocatalytic system, 2 pieces of the coated films (TiO_2_ film, Ag-TiO_2_ film, TiO_2_/H_3_PW_12_O_40_ composite film, or Ag-TiO_2_/H_3_PW_12_O_40_ composite film) with a weight of *ca*. 5.0 mg were submerged in the o-CP solution (100 ml). Prior to irradiation, the films were maintained in dark for 30 min to reach adsorption-desorption equilibrium of o-CP. After irradiation, a fixed amount of o-CP solution was sampled and analyzed at regular intervals. The degradation degree of o-CP solution was analyzed by HPLC equipped with Waters 2489 UV/visible detector and symmetry C18 (4.6 × 250 mm, particle size 5 μm), with a mobile phase of acetonitrile (40%) and H_2_O (60%, containing 0.1% acetic acid) at a flow rate of 0.7 ml·min^−1^ with a detection wavelength of 254 nm. The total organic carbon (TOC) was analyzed by a TOC-500 (Shimadzu). The intermediates during o-CP degradation were identified by a Waters Acquity UPLC/Quattro Premier XE LC/MS system. Besides, the concentrations of low molecular weight organic acids and Cl^−^ were tested using a DX-300 ion chromatography equipped with AS4A-SC column and CDM-II conductivity detector.

## Results and Discussion

### Characterization of the catalysts

#### ICP-AES and XPS

**Figure 1:**
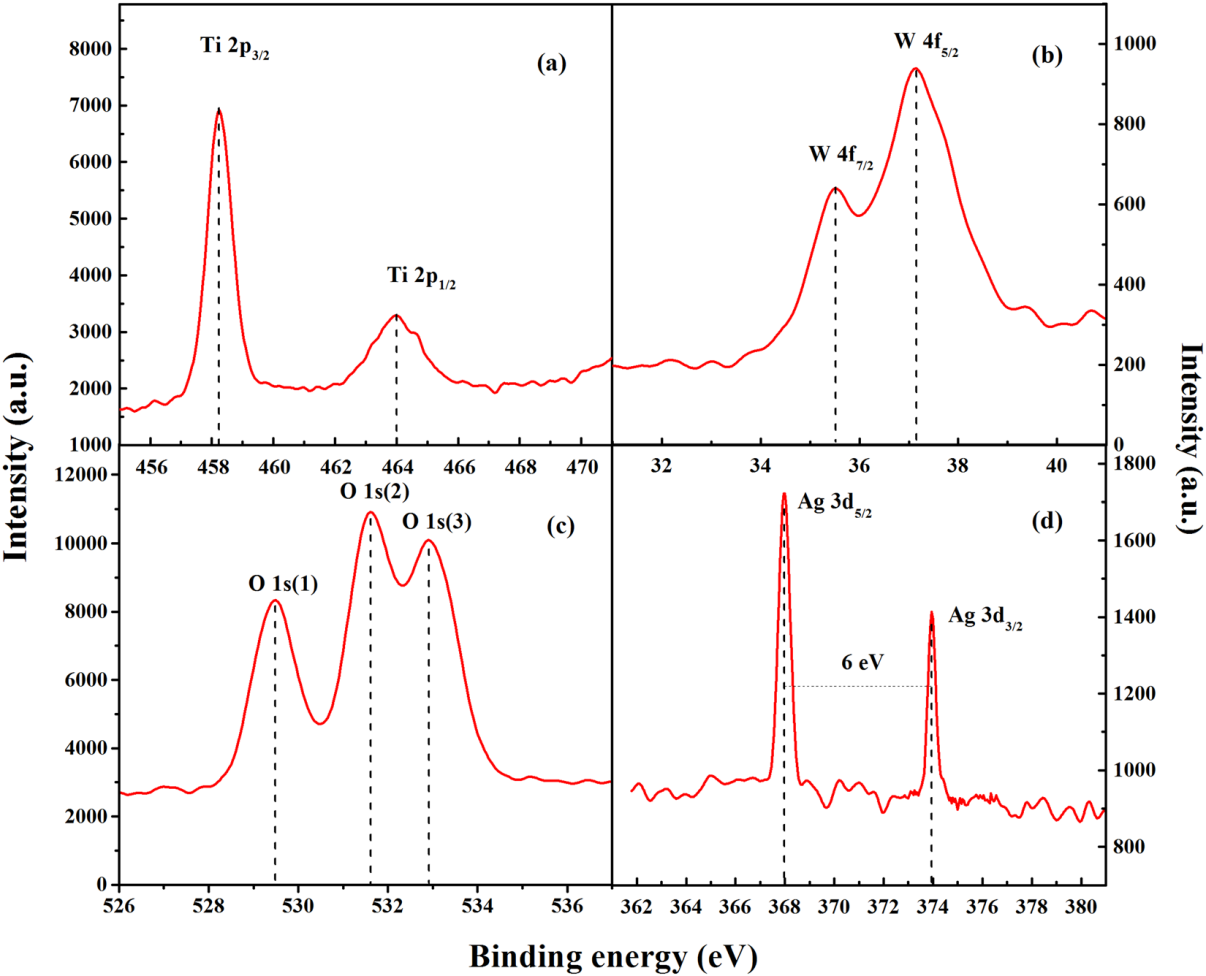
XPS survey spectra of the Ag-TiO_2_/H_3_PW_12_O_40_ film in the Ti 2p (**a**), W 4 f (**b**), O 1 s (**c**), and Ag 3d (**d**) binding energy regions.

The composition and structure of Ag-TiO_2_/H_3_PW_12_O_40_ composite films were informed by ICP-AES (Table 1) and XPS (Fig. 1), respectively. The loadings of H_3_PW_12_O_40_ and Ag in a serial of Ag-TiO_2_/H_3_PW_12_O_40_ composite films are listed in Table 1. The results indicated that Keggin unit and metallic Ag were successfully loaded by the current methods and the saturation Keggin structure of H_3_PW_12_O_40_ was retained integrally in the composite film with a P: W ratio of 1:12. Figure 1 shows that XPS spin-orbit lines of Ti 2p_3/2_ (458.3 eV), Ti 2p_1/2_ (464.0 eV), W 4f_7/2_ (35.5 eV), W 4f_5/2_ (37.2 eV), Ag 3d_5/2_ (368.0 eV) and Ag 3d_3/2_ (374.0 eV) were characteristic of Ti(IV) oxidation state, W(IV) oxidation state and metallic Ag in Ag-TiO_2_/H_3_PW_12_O_40_ composite film, respectively^29–31^. O 1 s XPS line of Ag-TiO_2_/H_3_PW_12_O_40_ composite film exhibited three peaks at 529.5 eV, 531.6 eV and 532.9 eV, originating from lattice oxygen species of TiO_2_, Keggin unit and adsorbed oxygen, respectively^44,45^. Thus, it can be concluded that (1) according to the location of Ti 2p_3/2_ and Ti 2p_1/2_, TiO_2_ was in anatase phase in Ag-TiO_2_/H_3_PW_12_O_40_ composite film^46^; (2) compared with pure TiO_2_ and H_3_PW_12_O_40_, both Ti 2p and W 4 f XPS lines of Ag-TiO_2_/H_3_PW_12_O_40_ composite film shifted toward low values, implying the formation of (≡Ti-OH_2_)_n_^+^[H_3-n_PW_12_O_40_]^n^ between Keggin unit and TiO_2_ support via Ti-O-W bond^12^; (3) the introduction of metallic Ag was confirmed by a spin energy separation of 6.0 eV^47^, since under heating parent AgNO_3_ was decomposed gradually into metallic Ag that tended to aggregate to form nanocrystals, as the following reactions^48^:

1 $${{\rm{AgNO}}}_{3}\mathop{\to }\limits^{{\rm{\Delta }}}{{\rm{Ag}}}_{2}{\rm{O}}+{{\rm{NO}}}_{2}+{\rm{NO}}$$

2 $${{\rm{Ag}}}_{2}{\rm{O}}\mathop{\to }\limits^{{\rm{\Delta }}}{\rm{Ag}}+{{\rm{O}}}_{2}$$

3 $${n}{{\rm{Ag}}}^{0}\to {({{\rm{Ag}}}^{0})}_{{n}}$$

**Table 1:** Actual loadings of H_3_PW_12_O_40_ and Ag in composite film.

The ternary composite films with different theory loadings	H_3_PW_12_O_40_ loading	Ag loading	P (μg·ml^−1^)	W (μg·ml^−1^)	P:W
Ag(0.5%)-TiO_2_/H_3_PW_12_O_40_(10%)	7.47%	0.23%	3.687	44.321	12.021
Ag(1%)-TiO_2_/H_3_PW_12_O_40_(10%)	7.43%	0.45%	3.621	43.502	12.014
Ag(2%)-TiO_2_/H_3_PW_12_O_40_(10%)	7.40%	1.27%	3.598	43.216	12.011
Ag(1%)-TiO_2_/H_3_PW_12_O_40_(5%)	3.32%	0.50%	1.478	17.720	11.989
Ag(1%)-TiO_2_/H_3_PW_12_O_40_(15%)	12.46%	0.40%	7.985	95.788	11.996

#### UV-Vis DRS

**Figure 2:**
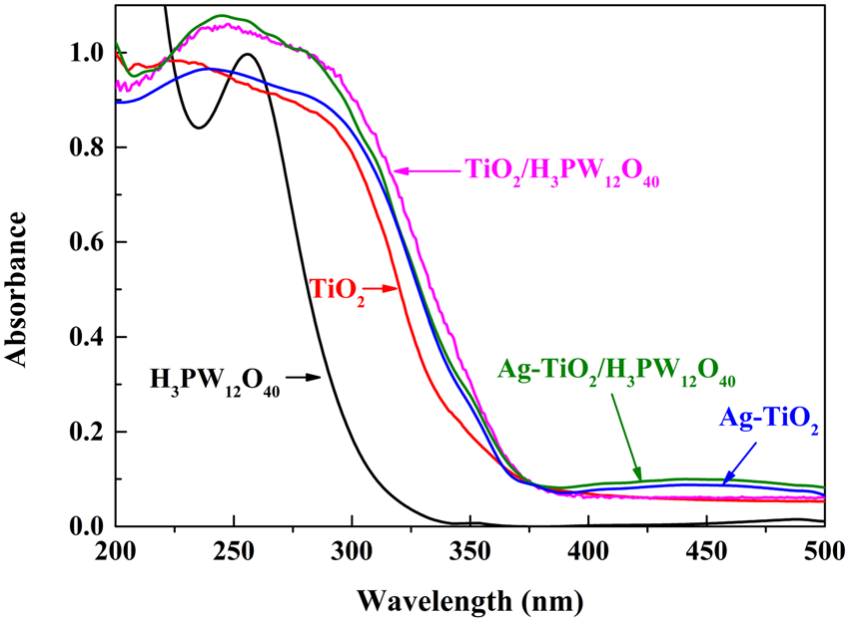
UV-Vis DRS of TiO_2_, H_3_PW_12_O_40_, Ag-TiO_2_, TiO_2_/H_3_PW_12_O_40_ and Ag-TiO_2_/H_3_PW_12_O_40_ film.

UV-Vis DRS analysis of TiO_2_, H_3_PW_12_O_40_, Ag-TiO_2_, TiO_2_/H_3_PW_12_O_40_ and Ag-TiO_2_/H_3_PW_12_O_40_ is illustrated in Fig. 2. Compared with TiO_2_ film, a broad and strong absorption was exhibited from 200 to 400 nm, which was contributed to the characteristic absorption peak of H_3_PW_12_O_40_; meanwhile a significant redshift of TiO_2_/H_3_PW_12_O_40_ film was observed, which was derived from the generation of narrow band gap through the hybridization of Ti 3d and W 5d orbit in TiO_2_/H_3_PW_12_O_40_ film^12^. It is worth noting that owing to SPR effect of metallic Ag^49^, both Ag-TiO_2_ and Ag-TiO_2_/H_3_PW_12_O_40_ composite film showed a light response in visible-light region (400–500 nm), which was superior to other modification methods^50,51^. Overall, the introduction of Ag extended the light response region and increased the light harvesting efficiency.

#### XRD and TEM

**Figure 3:**
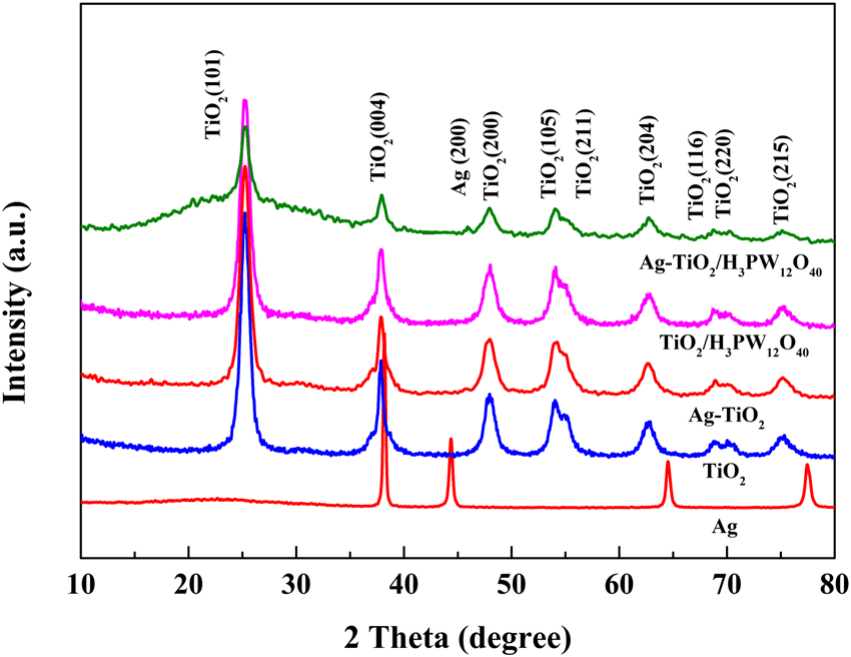
XRD patterns of TiO_2_, Ag, Ag-TiO_2_, TiO_2_/H_3_PW_12_O_40_ and Ag-TiO_2_/H_3_PW_12_O_40_ film.

Figure 3 presents XRD patterns of TiO_2_, Ag, Ag-TiO_2_, TiO_2_/H_3_PW_12_O_40_ and Ag-TiO_2_/H_3_PW_12_O_40_. The results indicated that all films showed well-indexed anatase phase of TiO_2_ (JCPDS 21–1272)^52^. The peaks at 38.1°, 44.3°, 64.4°, and 77.4° can be indexed to (111), (200), (220), and (311) diffractions of cubic structured Ag (JCPDS 65–2871), respectively^44^. It exhibited that Ag was loaded in cubic phase with the weak diffraction at 46.5° in both as-prepared Ag-TiO_2_ and Ag-TiO_2_/H_3_PW_12_O_40_ film. Meanwhile, compared with pure TiO_2_, the crystallinity of Ag-TiO_2_/H_3_PW_12_O_40_ composite film decreased, owing to the fine dispersion of Ag nanoparticles throughout the anatase lattice, which restricted the growth of crystallite.

**Figure 4:**
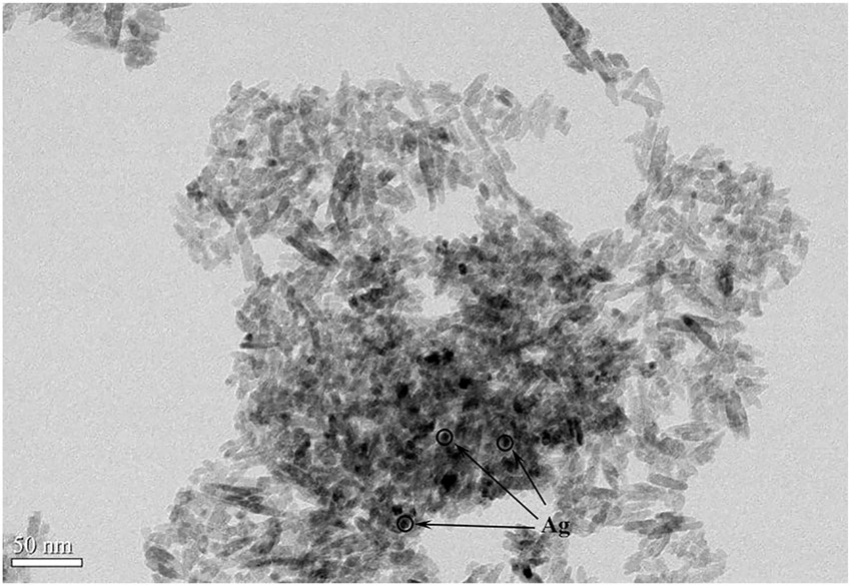
TEM image of Ag-TiO_2_/H_3_PW_12_O_40_ film.

**Figure 5:**
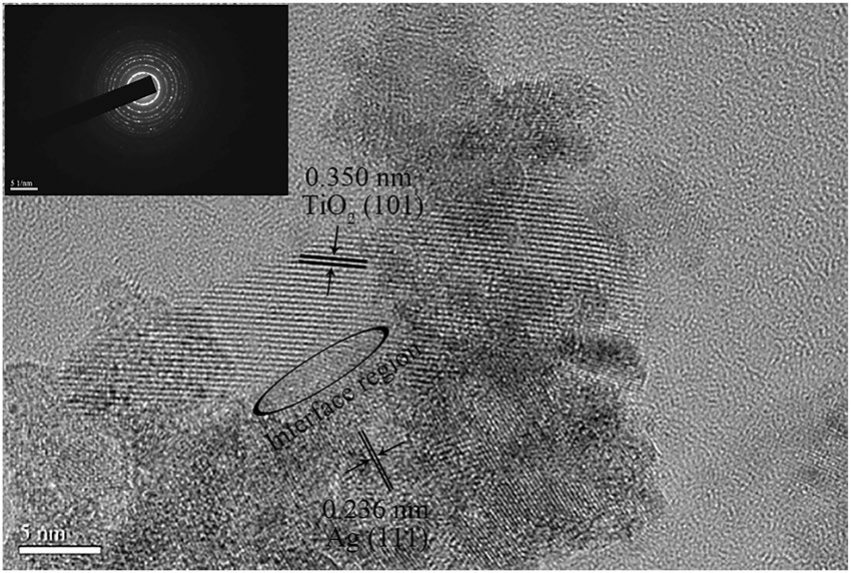
HRTEM image of Ag-TiO_2_/H_3_PW_12_O_40_ film (Inset: SEAD pattern).

Ag^0^ nanoparticles were well dispersed as the dark dots in TEM image (Fig. 4) and incorporated into Ag-TiO_2_/H_3_PW_12_O_40_ film. HRTEM image (Fig. 5) revealed a clear interface combination between the continuity of lattice fringes of TiO_2_ and metallic Ag nanoparticles indicating the formation of the heterojunction. At the interface, electrons flew from one material with the high-energy Fermi level to another with the low-energy Fermi level. Whereas, the Schottky barrier that formed at the heterojunction acted as an electron sink resulting in a depletion layer that maintained the charge separation^53–55^.

Both anatase TiO_2_ and cubic Ag crystallites were reconfirmed in the as-prepared Ag-TiO_2_/H_3_PW_12_O_40_ corresponding to the characteristic lattice fringe of 0.35 nm (101) and 0.236 nm (111), respectively. Furthermore, in SAED pattern of Ag-TiO_2_/H_3_PW_12_O_40_ film (inset of Fig. 5), a set of concentric rings corresponded to (101), (004), (200), (211), and (204) diffraction of anatase phase from the inner to the outer.

#### Raman

**Figure 6:**
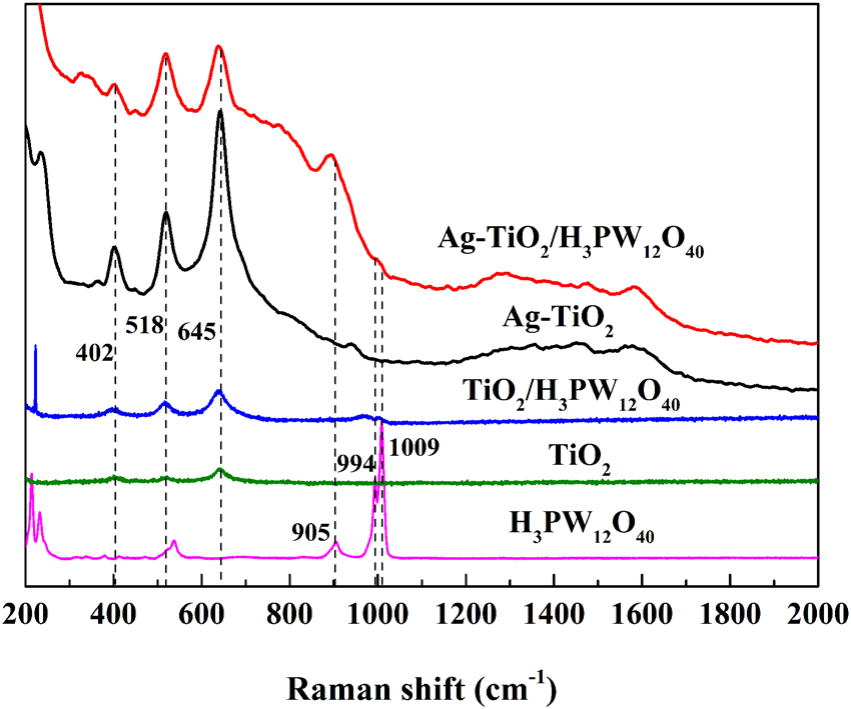
Raman spectra of H_3_PW_12_O_40_, TiO_2_, TiO_2_/H_3_PW_12_O_40_, Ag-TiO_2_ and Ag-TiO_2_/H_3_PW_12_O_40_ film.

The Raman spectra of H_3_PW_12_O_40_, TiO_2_, TiO_2_/H_3_PW_12_O_40_, Ag-TiO_2_ and Ag-TiO_2_/H_3_PW_12_O_40_ film were presented in Fig. 6. The peaks at 402 cm^−1^ (*B*_*1g*_), 518 cm^−1^ (*B*_*1g*_) and 645 cm^−1^ (*E*_*g*_) originated from anatase TiO_2_, and those at 905 cm^−1^, 994 cm^−1^, and 1009 cm^−1^ corresponded to stretching vibrations of W-O-W bonds, W = O bonds of Keggin unit and P-O bonds of PO_4_ units.

The surface-enhanced Raman scattering (SERS) signals indicated a strong SPR effect was generated in all the Ag-deposited films in spite of such a low Ag deposition amount. Compared with no Ag-deposited film, SPR excited by visible light would lead to an enhanced electromagnetic field around the nanoparticle, which could significantly promote the generation of “hot electron” at the interface of metal particle Ag and semiconductor TiO_2_. Furthermore, a potential energy difference (E_SPR_-φ_b_) between potential energy (E_SPR_) and Schottky barrier (φb) was also established at the interface according to the energy band structure feature of Ag and TiO_2_ crystal, which could facilitate the transfer of “hot electrons” from Ag to the conduction band (CB) of TiO_2_ and hinder the reverse transfer at the same time. While the shift of Raman peaks may be owing to the alterations of electronic density induced by electrons transfer among TiO_2_, H_3_PW_12_O_40_ and Ag, which may improve the activity of catalyst. It was also agreed with other reports^56,57^.

#### SEM

**Figure 7:**
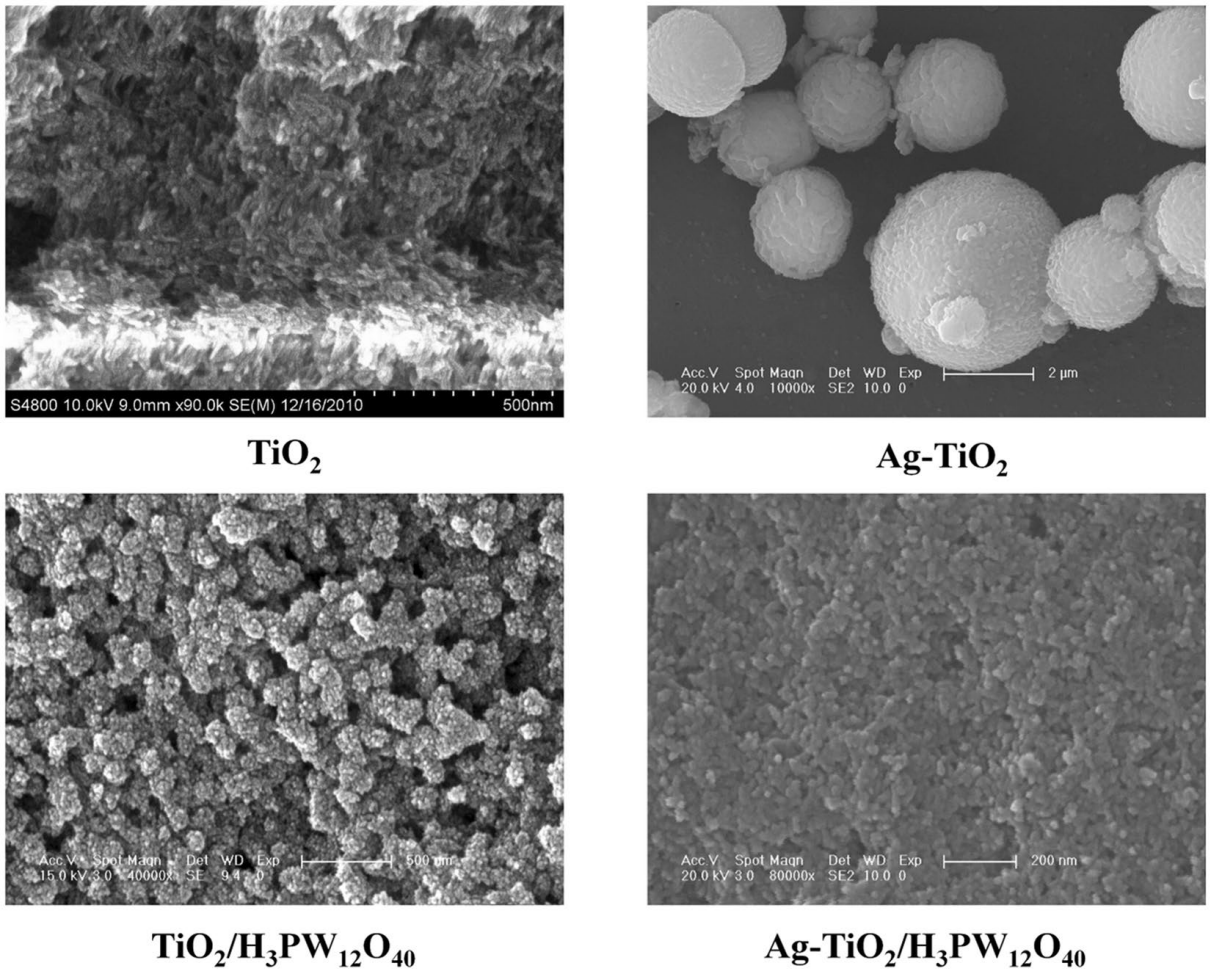
FESEM of TiO_2_, Ag-TiO_2_, TiO_2_/H_3_PW_12_O_40_, and Ag-TiO_2_/H_3_PW_12_O_40_ film.

**Figure 8:**
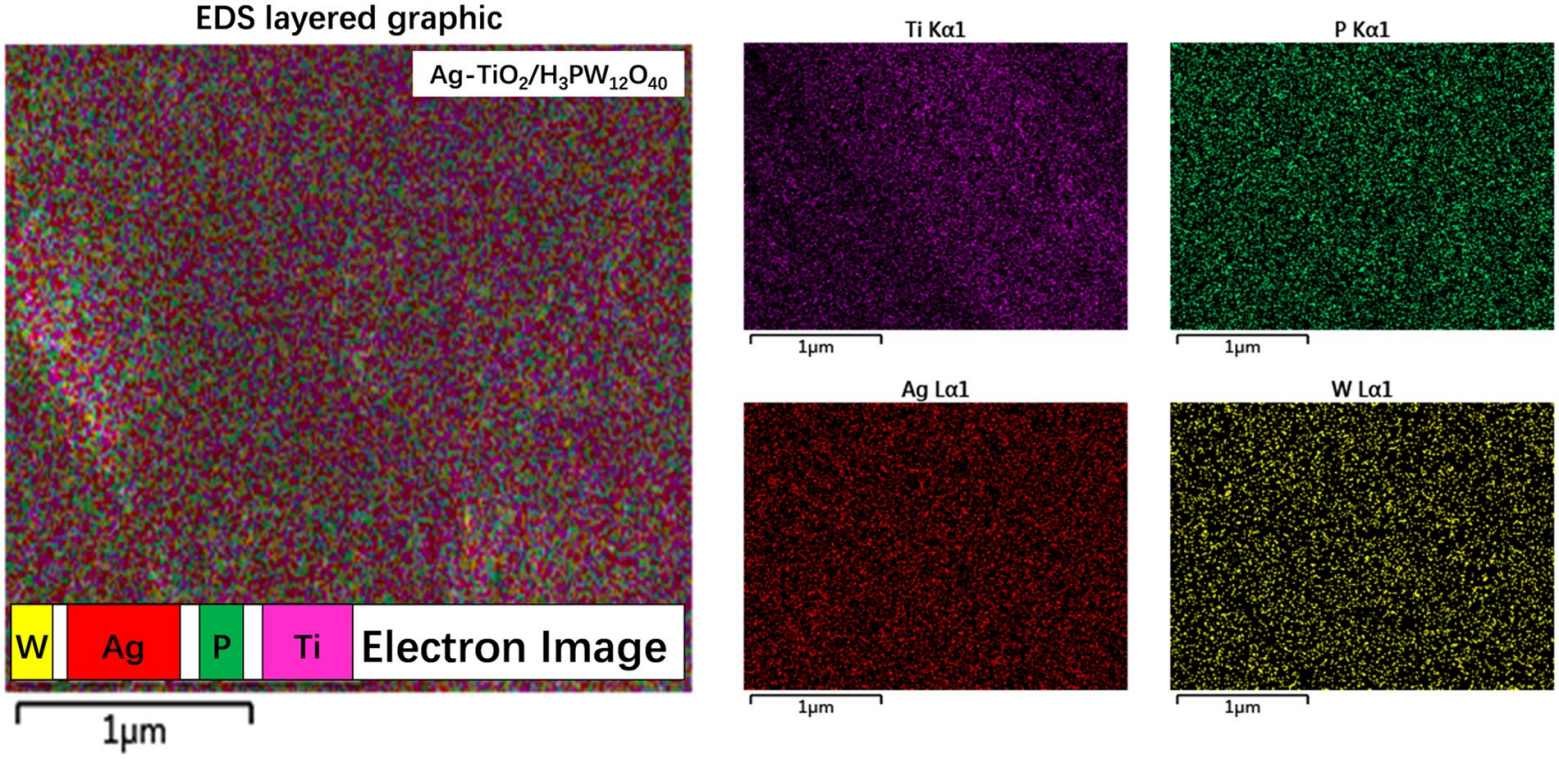
EDS mapping of TiO_2_, Ag-TiO_2_, TiO_2_/H_3_PW_12_O_40_, and Ag-TiO_2_/H_3_PW_12_O_40_ film (Ti: Violet, P: Green, Ag: Red, and W: Yellow).

As shown by FESEM (Fig. 7), the as-prepared TiO_2_, Ag-TiO_2_, TiO_2_/H_3_PW_12_O_40_, and Ag-TiO_2_/H_3_PW_12_O_40_ film varied considerably in morphology. TiO_2_ particles illustrated a regular rice shape with a size of *ca*. 80 nm; Ag-TiO_2_ particles were composed with spheres of TiO_2_ (2–4 μm) and Ag (ca. 500 nm); TiO_2_/H_3_PW_12_O_40_ particles displayed as spheres with diameters ranging between 80–100 nm covered by packed humps; whereas the surface of Ag-TiO_2_/H_3_PW_12_O_40_ particles became smoother than that of TiO_2_/H_3_PW_12_O_40_ due to the deposition of Ag into the pore structures of TiO_2_/H_3_PW_12_O_40_. Ti, P, W, and Ag were observed to distribute homogeneously in Ag-TiO_2_/H_3_PW_12_O_40_ film by EDS analysis (Fig. 8).

#### BET and BJH

**Figure 9:**
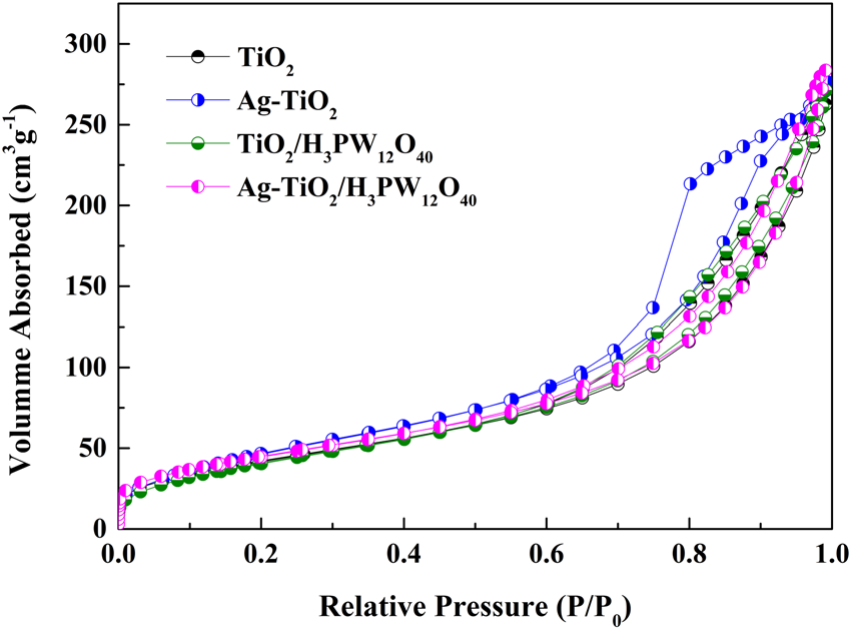
Nitrogen adsorption-desorption isotherms of TiO_2_, Ag-TiO_2_, TiO_2_/H_3_PW_12_O_40_ and Ag-TiO_2_/H_3_PW_12_O_40._

**Figure 10:**
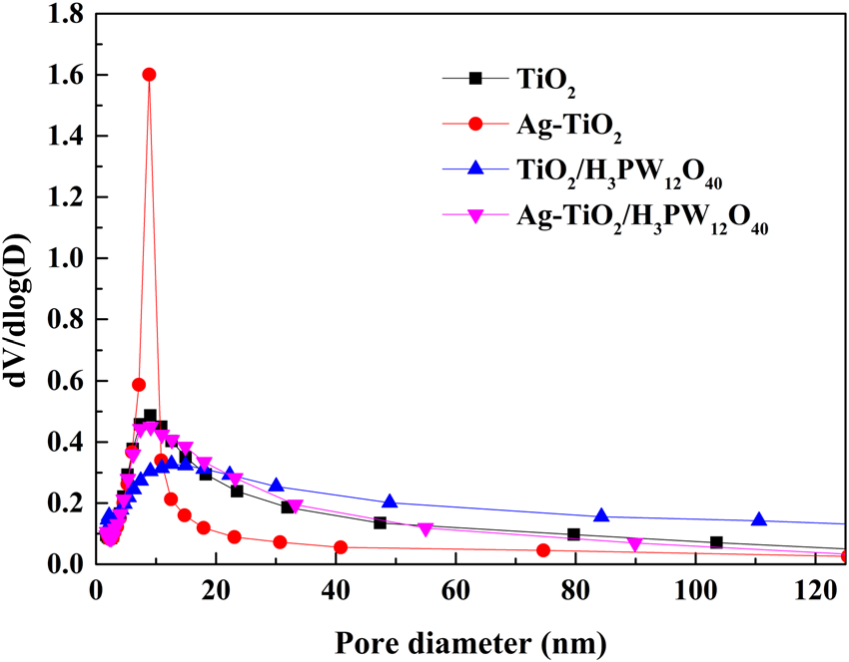
Pore size distribution profiles of TiO_2_, Ag-TiO_2_, TiO_2_/H_3_PW_12_O_40_ and Ag-TiO_2_/H_3_PW_12_O_40_.

Figure 9 exhibits the adsorbed nitrogen amounts increased rapidly at p/p_0_ < 0.1 and H3 hysteresis loop excited at p/p_0_ = 0.4–0.8, indicating the presence of microporosity (<2 nm) and mesoporosity (2–50 nm) in the prepared photocatalysts (TiO_2_, Ag-TiO_2_, TiO_2_/H_3_PW_12_O_40_ and Ag-TiO_2_/H_3_PW_12_O_40_). BET surface area and pore volume of each prepared photocatalyst are summarized in Table 2. These results were consistent with BJH desorption pore distribution curves and pore diameters calculated by BJH method (Fig. 10). BET surface area (169.9 m^2^·g^−1^) and pore volume (0.4390 cm^3^·g^−1^) of TiO_2_/H_3_PW_12_O_40_ was higher than those of TiO_2_ (158.6 m^2^·g^−1^; 0.4240 cm^3^·g^−1^) due to the formation of TiO_2_/H_3_PW_12_O_40_ framework via Ti-O-W bonds. Whereas, after depositing of Ag, BET surface area and pore volume decreased to 159.1 m^2^·g^−1^ and 0.4256 cm^3^·g^−1^, respectively. It can be concluded that Ag may be inserted into the pore structures of TiO_2_/H_3_PW_12_O_40_, which enhanced the stability of metallic Ag^32^. In spite of this, BET surface area of Ag-TiO_2_/H_3_PW_12_O_40_ was much higher than that of commercial TiO_2_ P25 (50 m^2^·g^−1^).

**Table 2:** The BET surface area and pore volume of the catalysts.

Sample	S_BET_ (m^2^·g^−1^)	V_p_ (cm^3^·g^−1^)
TiO_2_	158.6	0.4240
Ag-TiO_2_	182.2	0.4985
TiO_2_/H_3_PW_12_O_40_	169.9	0.4390
Ag-TiO_2_/H_3_PW_12_O_40_	159.1	0.4256
Commercial TiO_2_ P25	50	—

### Photocatalytic test

As a kind of persistent organic pollutant and priority pollutant listed by US EPA, o-CP was employed to evaluate the photocatalytic activity of as-prepared Ag-TiO_2_/H_3_PW_12_O_40_ composite film under the simulated sunlight, together with TiO_2_, Ag-TiO_2_ and TiO_2_/H_3_PW_12_O_40_ as comparisons. Moreover, the influencing factors were also investigated including the loading amounts of H_3_PW_12_O_40_ and Ag, initial o-CP concentration, initial pH value as well as the kinetics of o-CP photodegradation.

### Comparison of photocatalytic activity among TiO_2_, Ag-TiO_2_, TiO_2_/H_3_PW_12_O_40_ and Ag-TiO_2_/H_3_PW_12_O_40_

**Figure 11:**
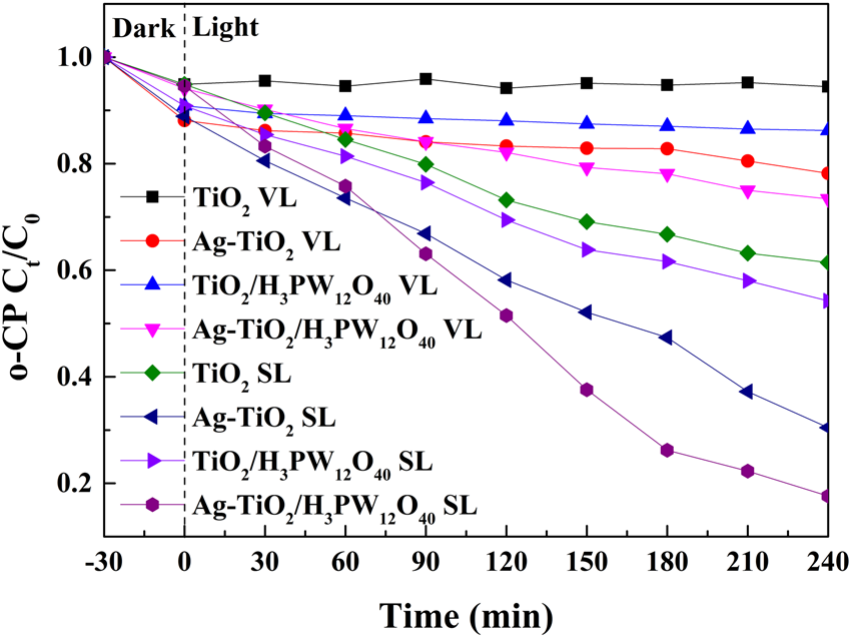
The photocatalytic activity of TiO_2_, Ag-TiO_2_, TiO_2_/H_3_PW_12_O_40_ and Ag-TiO_2_/H_3_PW_12_O_40_ towards o-CP degradation. (VL: Visible light; SL: Simulated sunlight).

Figure 11 illustrates the photocatalytic degradation of o-CP (5 mg·L^−1^, 100 ml, pH = 6.3) by different catalysts under simulated sunlight irradiation. After 4 h irradiation, the degradation efficiency of o-CP was 82.40% by Ag-TiO_2_/H_3_PW_12_O_40_ composite film, 69.55% by Ag-TiO_2_ composite film, 45.75% by TiO_2_/H_3_PW_12_O_40_ composite film, and 38.55% by TiO_2_ film. H_3_PW_12_O_40_ improved the photocatalytic activity of TiO_2_, owing to the synergistic effects between Keggin unit and TiO_2_, which hindered the recombination of h^+^-e^−^ pairs efficiently^12,13^. Additionally, loading of Ag could not only enhance the quantum efficiency via generating Schottky junction at the interface between Ag and TiO_2_ (Fig. 5), but also increase the absorption of visible-light due to SPR effect, which has been confirmed by the above characterization (Fig. 2).

In order to clarify the enhancement of SPR effect, o-CP photocatalytic degradation was carried out under visible light (The lamp was equipped with the filter to cut UV light with 200–400 nm wavelength). After introducing Ag into TiO_2_ and TiO_2_/H_3_PW_12_O_40_ film, the degradation efficiency towards o-CP increased from 5.55% (TiO_2_) to 21.80% (Ag-TiO_2_), and from 13.75% (TiO_2_/H_3_PW_12_O_40_) to 26.60% (Ag-TiO_2_/H_3_PW_12_O_40_) (Fig. 11), which was attributed to the loading of plasmonic metal.

The adsorption capacity of all the photocatalysts (Fig. S1 of Supporting Information) was limited (TiO_2_: 6.67%; Ag-TiO_2_: 12.38%; TiO_2_/H_3_PW_12_O_40_: 9.94%; Ag-TiO_2_/H_3_PW_12_O_40_: 5.89%) even though they possessed large BET surface area, which could be attributed to the low amount of the catalyst (*ca*. 5.0 mg) in the current system. Compared with other related researches, the as-prepared Ag-TiO_2_/H_3_PW_12_O_40_ composite film represented a more excellent property on the light utilization with a comparable catalyst amount^58^.

### Effect of H_3_PW_12_O_40_ loading amount

**Figure 12:**
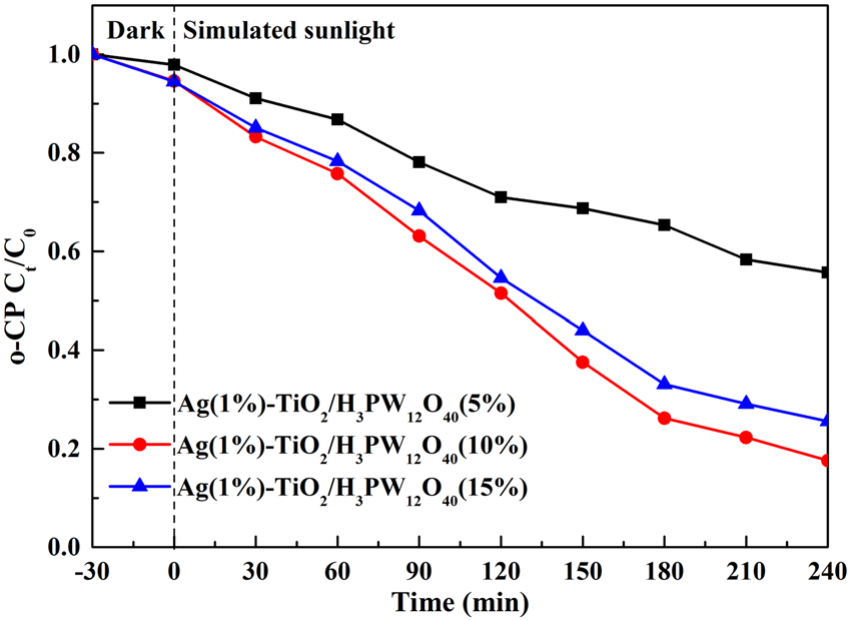
The influence of H_3_PW_12_O_40_ loading amount on the photocatalytic activity of Ag-TiO_2_/H_3_PW_12_O_40_ towards o-CP degradation.

The adsorption of o-CP (5 mg·L^−1^, 100 ml, pH = 6.3) on Ag(1%)-TiO_2_/H_3_PW_12_O_40_ with different H_3_PW_12_O_40_ loading amount is shown in Fig. S2. The results suggested that H_3_PW_12_O_40_ loading amount did not exert a significant influence on adsorption capacity to o-CP. Figure 12 exhibits the effect of H_3_PW_12_O_40_ loading amount on the photocatalyic activity of Ag(1%)-TiO_2_/H_3_PW_12_O_40_ film. The photocatalytic activity raised obviously with the increase of H_3_PW_12_O_40_ loading amount from 5% to 10%, whereas it decreased slightly when H_3_PW_12_O_40_ loading reached 15%, since excessive amount of H_3_PW_12_O_40_ might affect light absorption^11^. Consequently, the degradation rate peaked at 82.40% by Ag(1%)-TiO_2_/H_3_PW_12_O_40_(10%).

### Effect of Ag loading amount

**Figure 13:**
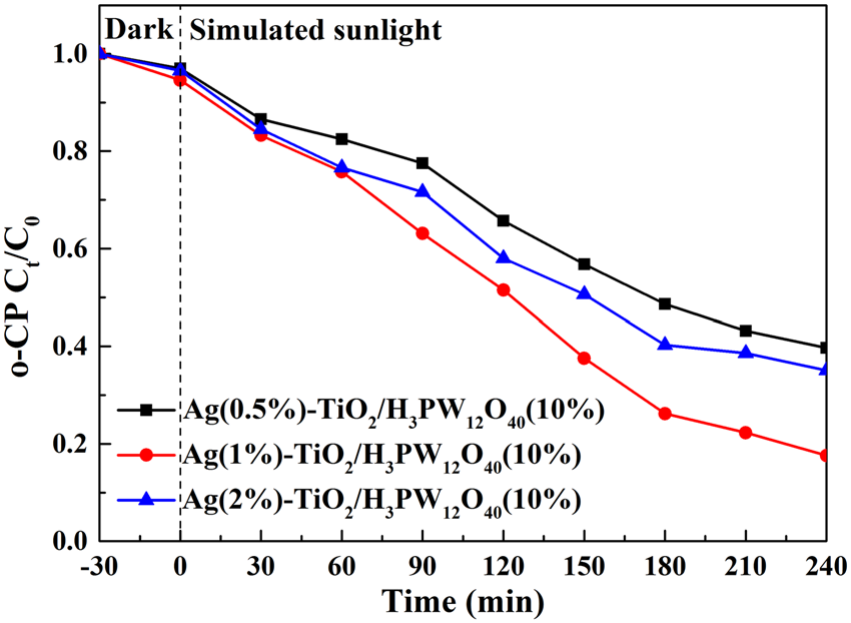
The influence of Ag loading amount on the photocatalytic activity of Ag-TiO_2_/H_3_PW_12_O_40_ towards o-CP degradation.

Likewise, the variation of Ag loading amount in Ag-TiO_2_/H_3_PW_12_O_40_(10%) films did not alter the adsorption capability of o-CP (5 mg·L^−1^, 100 ml, pH = 6.3) significantly (Fig. S3). The degradation efficiency of o-CP (82.40%) peaked with a Ag loading amount of 1% in Ag-TiO_2_/H_3_PW_12_O_40_ film (Fig. 13). It implied that a great majority of transferred electrons were trapped due to the strong electron accepting ability of metallic Ag, resulting in an effective separation of the electrons and holes. However, excessive Ag nanoparticle not only acted as an electron-hole recombination center, but also blocked partial UV-light that could reach the surface of TiO_2_^59,60^, which further decreased its photocatalytic activity. Thus, Ag(1%)-TiO_2_/H_3_PW_12_O_40_(10%) represented the maximum photoactivity, and was selected in subsequent photocatalytic experiments.

### Effect of initial concentration of o-CP

**Figure 14:**
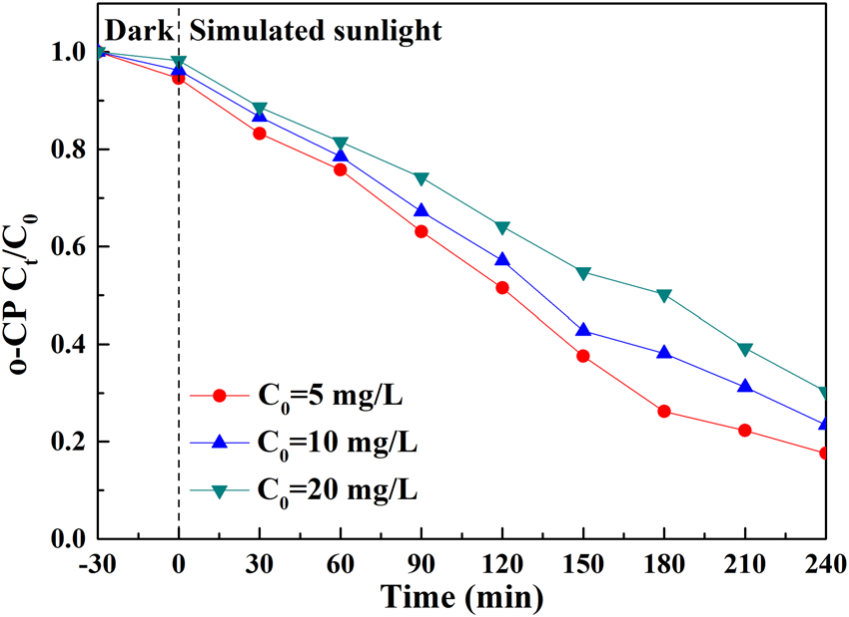
The influence of initial concentration of o-CP on the photocatalytic activity of Ag-TiO_2_/H_3_PW_12_O_40_.

As demonstrated in Fig. S4, the direct photolysis rate of o-CP (100 ml, pH = 6.3) was 5.11%, 3.83% and 1.83% with initial concentrations of 5 mg·L^−1^, 10 mg·L^−1^, and 20 mg·L^−1^, the photocatalytic degradation rate was 82.40%, 76.60% and 63.70%, respectively (Fig. 14). Thus, both the direct photolysis and photocatalytic degradation rate decreased gradually with the raise of initial concentration of o-CP by reason of the restraint on light transmittance and light utilization of catalyst. Additionally, with a fixed catalyst dosage, the more o-CP molecules adsorbed and accumulated on the film surface (Fig. S5), the less contact between the reactive oxygen species and catalyst^61^. Herein, the minimum initial concentration (5 mg·L^−1^) was the optimal condition for photocatalytic degradation.

### Effect of initial pH

**Figure 15:**
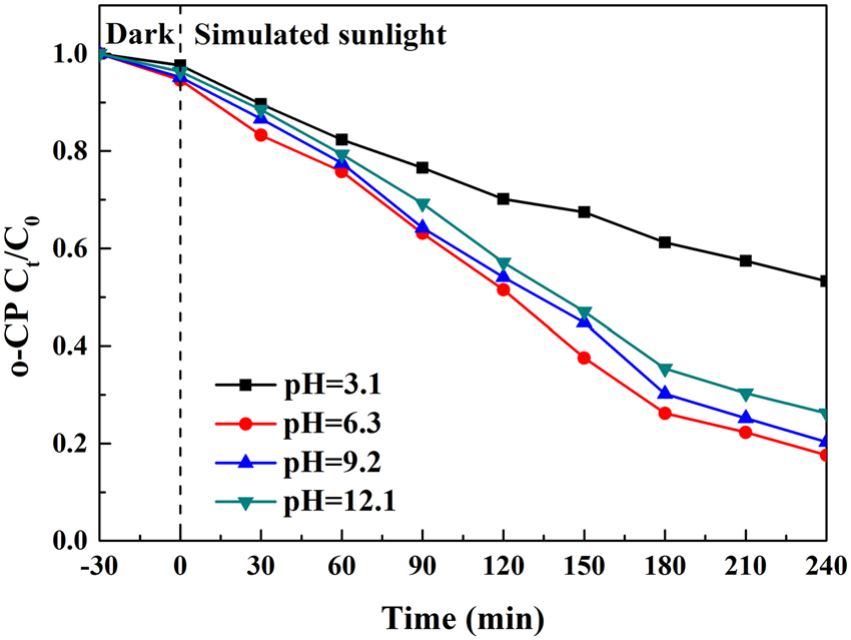
Influence of initial pH values towards o-CP degradation.

As illustrated in Fig. 15, at the alkaline conditions, the degradation rate was 79.70% at pH = 9.2 and 73.80% at pH = 12.1, which was significantly higher than that at the acid condition (46.80% at pH = 3.1). The contact of o-CP molecules with the catalyst or sunlight irradiation was intercepted under the acid condition (Figs S6 and S7), resulting in a low degradation efficiency. Under the alkaline condition, the adsorption process was hindered by the electrostatic repulsion between the electronegative composite film (both Ag and H_3_PW_12_O_40_ are fairly strong electron acceptors) and negatively charged o-CP. Whereas, the direct photodegradation rate elevated rapidly with increase of pH values, since high pH value was in favor of generation of hydroxyl ions^62^, which would subsequently enhance the photodegradation efficiency via forming hydroxyl radicals with holes.

The photocatalytic degradation rate achieved the maximum value (82.40%) at pH = 6.3 due to the largest adsorption amount of o-CP. In addition, photocatalytic activity of TiO_2_ peaked at pH_pzc_ (pH = 6.25), which was close to the initial pH value of o-CP solution (pH = 6.3). Hence, pH = 6.3 was optimal initial pH value for the photodegradation of o-CP^63,64^.

### Photocatalytic kinetics

The kinetics of photocatalytic reactions under different conditions are summarized in Table 3. The results indicated the kinetics could be well described by simplified Langmuir-Hinshelhood (L-H) Model:

4 $$-dc/dt={K}_{app}c$$

in which *K*_*app*_ is the apparent constant as the basic kinetic parameter when the initial concentration is low; *c* is the initial concentration of the target compound.

**Table 3:** The kinetics of o-CP photocatalytic reaction.

Influence factor	Condition	*r*_0_ (mg/L·min)	*k* (min^−1^)	Kinetic equation	*R*
Film type	TiO_2_	0.0095	0.0019	y = 0.0019x + 0.0619	0.990
	Ag-TiO_2_	0.0230	0.0046	y = 0.0046x + 0.0136	0.990
	TiO_2_/H_3_PW_12_O_40_	0.0115	0.0023	y = 0.0023x + 0.0782	0.994
	Ag-TiO_2_/H_3_PW_12_O_40_	0.0377	0.0075	y = 0.0075x − 0.1095	0.990
H_3_PW_12_O_40_ loading	5%	0.0122	0.0024	y = 0.0024x + 0.0139	0.995
	10%	0.0377	0.0075	y = 0.0075x − 0.1095	0.990
	15%	0.0301	0.0060	y = 0.0060x − 0.0648	0.992
Ag loading	0.5%	0.0199	0.0040	y = 0.0040x − 0.0253	0.993
	1%	0.0377	0.0075	y = 0.0075x − 0.1095	0.990
	2%	0.0227	0.0045	y = 0.0045x + 0.0079	0.993
Initial pH	3.1	0.0127	0.0025	y = 0.0025x + 0.0278	0.997
	6.3	0.0377	0.0075	y = 0.0075x − 0.1095	0.990
	9.2	0.0343	0.0069	y = 0.0069x − 0.1077	0.989
	12.1	0.0294	0.0059	y = 0.0059x − 0.0815	0.993
Initial concentration	5 mg/L	0.0377	0.0075	y = 0.0075x − 0.1095	0.990
	10 mg/L	0.0236	0.0047	y = 0.0047x − 0.0677	0.984
	20 mg/L	0.0299	0.0040	y = 0.0060x − 0.0766	0.992

Under the optimal condition, *K*_*app*_ of o-CP photocatalytic degradation reaction achieved 0.0075 min^−1^ by Ag(1%)-TiO_2_/H_3_PW_12_O_40_(10%) film, which was 1.63-fold, 3.26-fold and 3.95-fold larger than that of Ag-TiO_2_, TiO_2_/H_3_PW_12_O_40_ film and TiO_2_ film, respectively. *K*_*app*_ fluctuated largely along with the variation of H_3_PW_12_O_40_ loading amount and initial pH value, which suggested both of the factors exerted an essential influence on the kinetics of o-CP degradation.

### Photocatalytic Mechanism

**Figure 16:**
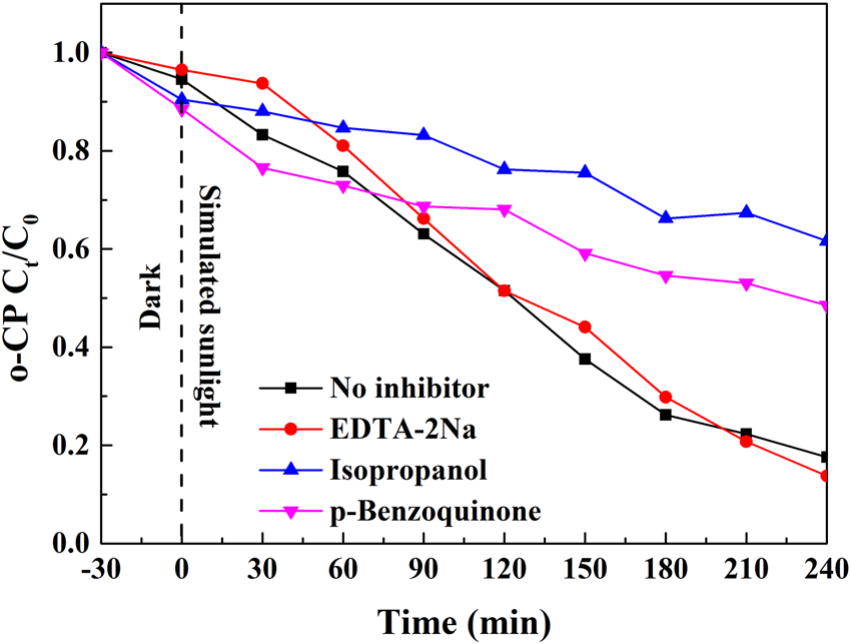
The degradation of o-CP by Ag-TiO_2_/H_3_PW_12_O_40_ with different scavengers. (**a**) Na_2_-EDTA (0.0037 g); (**b**) isopropanol (0.1 ml); (**c**) benzoquinone (0.0108 g).

In general, the photocatalytic degradation can be regarded as a process of generation, transfer, and consumption of the photogenerated carriers^65^. The photocatalyst absorbed the incident photons with energy above the semiconductor’s band gap, generating the same number of electrons and holes, in which the hole abstracted electrons from absorbed pollutants or reacted with H_2_O to generate ·OH; while the conduction band electrons reduced the absorbed oxygen to produce ·O_2_^−^ that further generated ·OH via chain reactions. In order to reveal the mechanism of enhanced photocatalytic activity of the plasmonic Ag-TiO_2_/H_3_PW_12_O_40_ photocatalyst in depth, the active species generated during the process of photocatalyzed o-CP degradation were identified by free radicals and holes trapping experiments in the current study. Na_2_-EDTA (0.0037 g)^66^, isopropanol (0.1 ml)^67^, and benzoquinone (0.0108 g)^68^ were employed to scavenge the holes (h^+^), hydroxyl radicals (·OH), and superoxide radicals (·O_2_^−^), respectively. After adding Na_2_-EDTA, the degradation efficiency did not alter significantly, implying the holes played a minor role in either oxidization or generation of ·OH during the o-CP degradation process. Whereas, the presence of isopropanol and benzoquinone decreased the degradation rate markedly to 38.4% and 51.5%, respectively, indicating both ·OH and ·O_2_^−^ acted as a major role during the process (Fig. 16).

**Figure 17:**
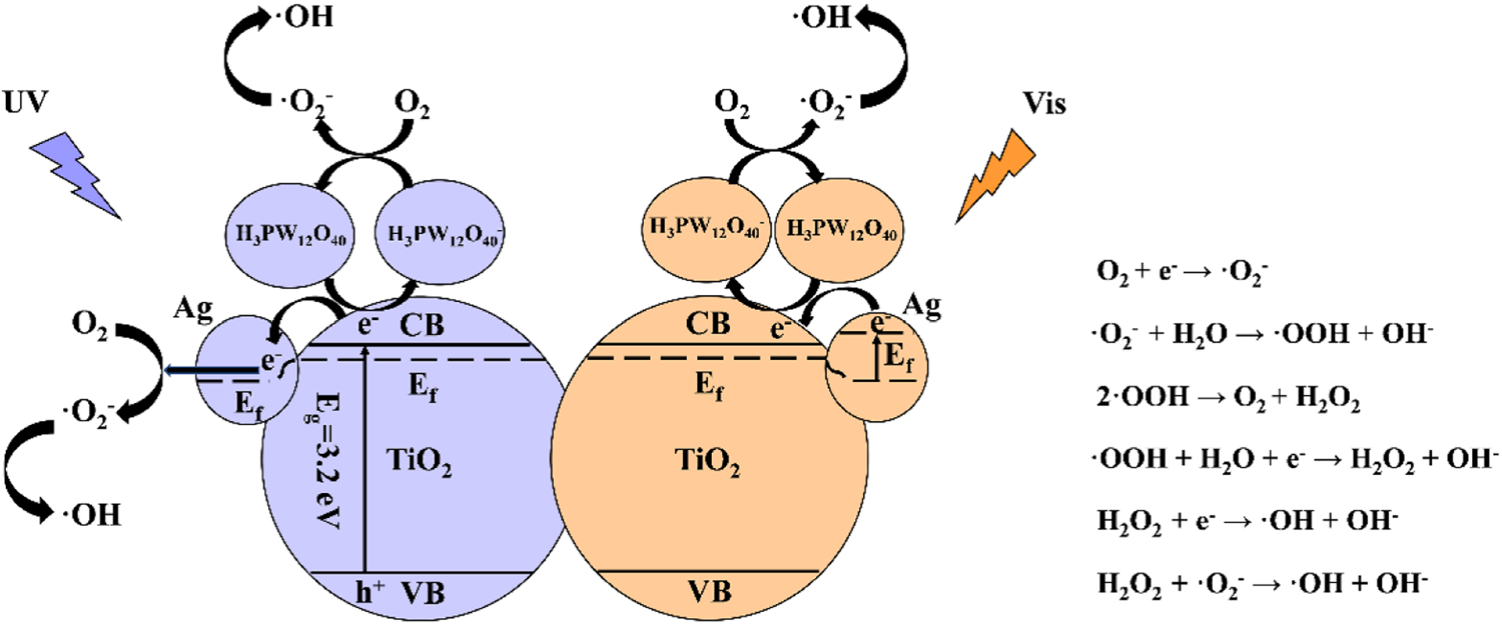
The photocatalytic mechanism of Ag-TiO_2_/H_3_PW_12_O_40_ system.

The detailed photocatalytic mechanism of Ag-TiO_2_/H_3_PW_12_O_40_ towards o-CP degradation under the simulated sunlight (320 nm < λ < 780 nm) is illustrated in Fig. 17. Under the UV-light (320 nm < λ < 400 nm), the electrons were firstly promoted from the valence band to the conduction band of TiO_2_, left the holes in the valence band of TiO_2_. Whereafter, the photogenerated electrons were transported constantly to metallic Ag and accumulated on its surface, forming the Schottky junction between Ag and TiO_2_. Furthermore, H_3_PW_12_O_40_ trapped the electrons which promoted to the conduction band of TiO_2_ generating H_3_PW_12_O_40_^−^, and then self-regenerated to H_3_PW_12_O_40_ via the redox cycling. Finally, these effectively separated electrons could be transferred to the oxygen absorbed on the surface of Ag and H_3_PW_12_O_40_ to generate ·O_2_^−^ active groups. Under the visible light (400 nm < λ < 780 nm), Ag was activated at first, the hot plasmonic electrons of Ag was subsequently transformed to the conduction band of TiO_2_ and gathered by H_3_PW_12_O_40_, consequently, the generated H_3_PW_12_O_40_^−^ reacted with adsorbed oxygen to produce ·O_2_^−^ which would further react with H_2_O to form·OH. To sum up, both ·O_2_^−^ and ·OH played a primary active role in the degradation of o-CP.

### Mineralization of o-CP

**Figure 18:**
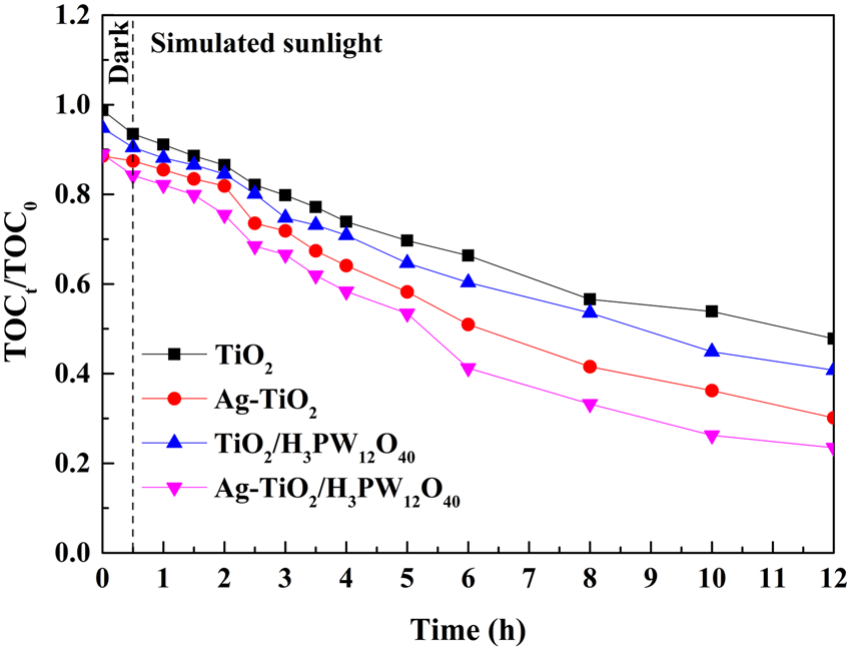
Variation of TOC during photocatalytic degradation of o-CP.

The mineralization capability of Ag-TiO_2_/H_3_PW_12_O_40_ film to o-CP molecules was evaluated by monitoring the variation of TOC in the reaction system during photocatalytic degradation process (Fig. 18). In order to detect it sensitively and accurately, the initial o-CP concentration was increased to 20 mg·L^−1^. The results indicated that in comparison with TiO_2_/H_3_PW_12_O_40_ (52.20%), Ag-TiO_2_ (69.88%) and TiO_2_ (49.20%) film, Ag-TiO_2_/H_3_PW_12_O_40_ film exhibited the highest mineralization capability, by which 76.50% of TOC was mineralized after 12 h irradiation, indicating that most of o-CP molecules as well as the organic intermediates were decomposed and mineralized.

**Figure 19:**
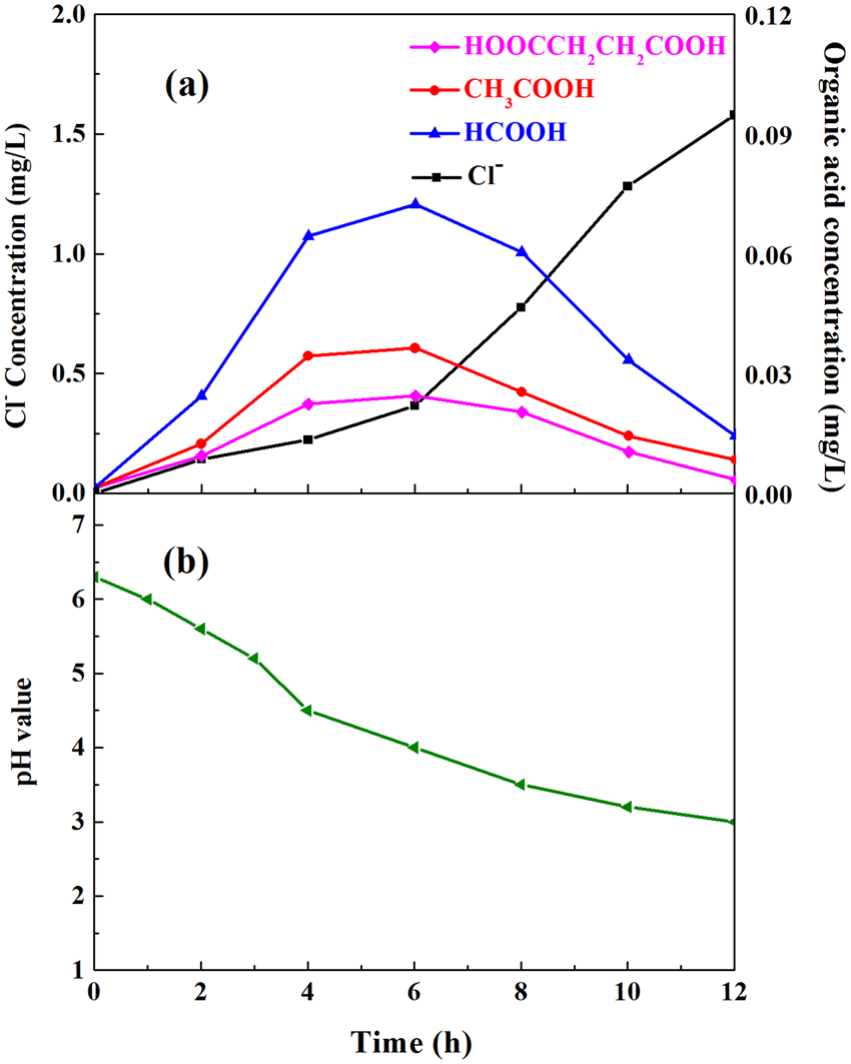
Evolution of low molecular weight organic acids and chlorine (**a**) and pH value (**b**) during photocatalytic degradation of o-CP.

Figure 19(a) shows that the concentrations of acetic acid and butanedioic acid peaked within 4–6 h during the degradation process, while the formic acid concentration achieved the maximum value within 4–8 h, suggesting the ring-opening reaction occurred during the o-CP degradation. The releasing rate of Cl^−^ was low before 6 h and increased greatly after 6 h, due to the occurrence of C-Cl bond cleavage. The results implied a possibility that the ring-opening reaction of o-CP molecule mainly occurred in the early stage during the degradation process, while most of C-Cl bond were broken subsequently. At 12 h, the concentration of Cl^−^ reached 1.58 mg·L^−1^, while the concentrations of low molecular weight organic acids decreased to 0.007 mg·L^−1^, 0.002 mg·L^−1^ and 0.013 mg·L^−1^ for acetic acid, butanedioic acid and formic acid, respectively, which could be further mineralized to CO_2_ and H_2_O. Moreover, as shown in Fig. 19(b), the releasing of Cl^−^ and formation of low molecular weight organic acids could decrease pH value during the process, which would impede the progress of photocatalytic degradation, as confirmed previously (Fig. 15). Therefore, the decrease of pH value during the degradation process may explain why o-CP cannot be decomposed and mineralized completely.

### Degradation pathways of o-CP

**Figure 20:**
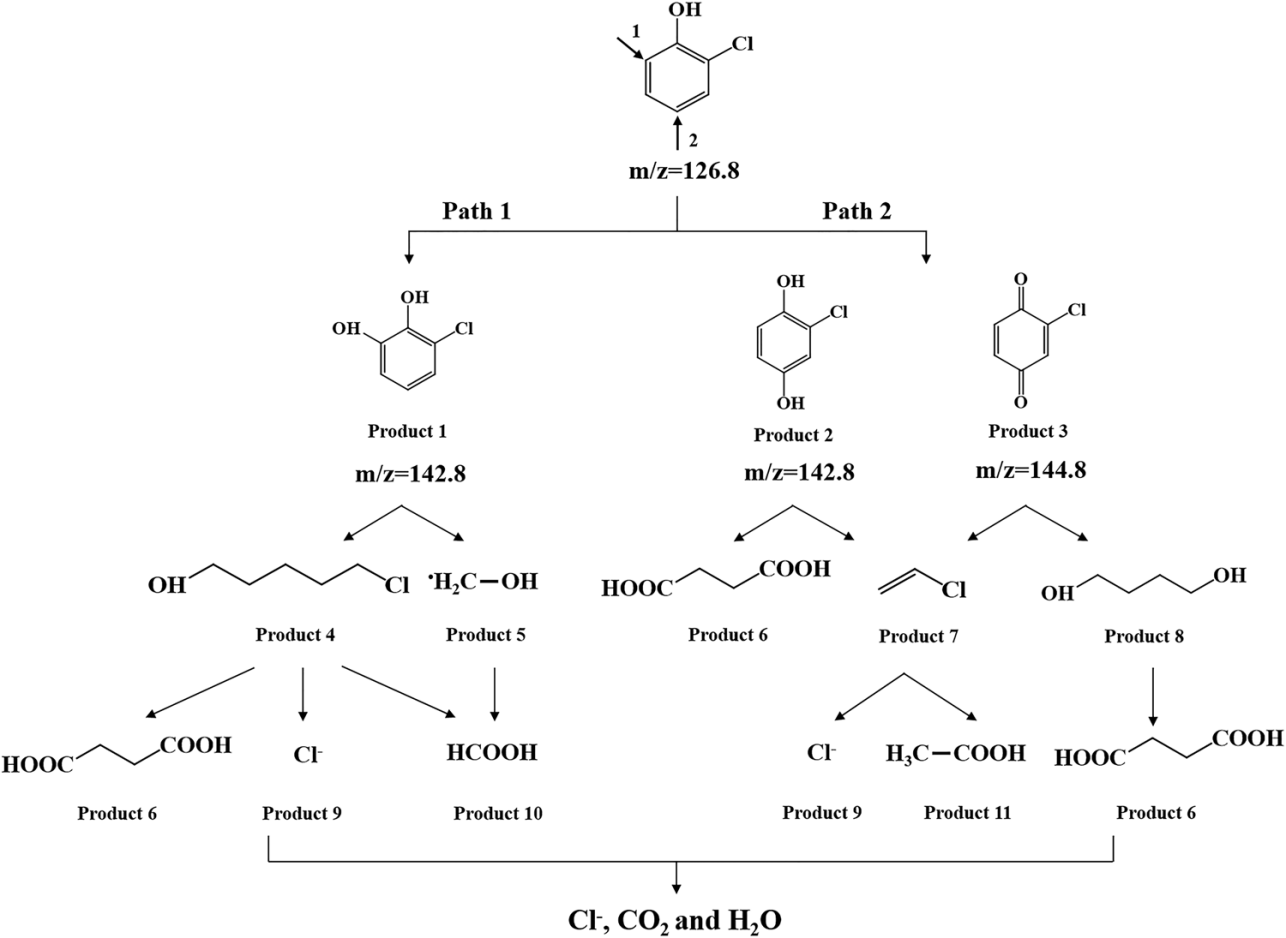
The possible photocatalytic degradation pathways of o-CP by Ag-TiO_2_/H_3_PW_12_O_40_ film.

In order to speculate the degradation pathways of o-CP in Ag-TiO_2_/H_3_PW_12_O_40_ film system, the main intermediate products were detected by LC-MS (Table 4). The mass fragment peaks were identified as *o*-chlorophenol (126.8 m/z), 2-chlorohydroquinone or 3-chlorocatechol (142.8 m/z), and 2-chlorobenzoquinone (144.8 m/z). Accordingly, the possible photocatalytic degradation pathways of o-CP were as follows (Fig. 20). As the key role in the photocatalytic degradation, ·OH and ·O_2_^−^ attacked preferentially the *ortho* and *para* position of o-CP molecule^69^. The *ortho* position (Path 1) was attacked by ·OH generating 3-chlorocatechol followed by H-abstraction, and then 5-chloropentanol was generated and further decomposed to formic acid, butanedioic acid and Cl^−^ after ring-opening reaction; The *para* position (Path 2) was attacked by both ·OH and ·O_2_^−^ producing 2-chlorohydroquinone and 2-chlorobenzoquinone simultaneously, hereafter, butanediol and butanedioic acid were formed via ring-opening reaction, together with chloroethylene as another intermediate product that further produced acetic acid by dechlorinating processes. Finally, o-CP can be mineralized into Cl^−^, CO_2_ and H_2_O.

**Table 4:** The chemical formulas of o-CP and the main intermediate products.

	m/z	Structural formula
o-CP	126.8	
Main intermediate products	142.8	
	144.8

### Recyclability of the catalyst

**Figure 21:**
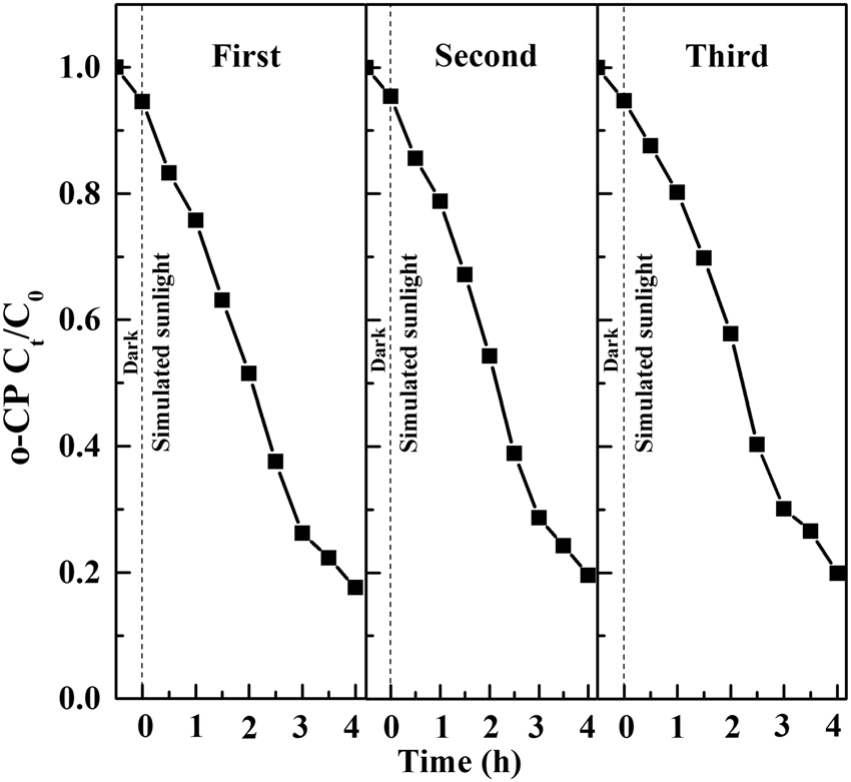
Recycling runs of Ag-TiO_2_/H_3_PW_12_O_40_ film in the photocatalytic degradation of o-CP.

From viewpoint of practical applications, the recyclability is an essential aspect for the composite film photocatalyst, which can not only greatly reduce the cost but also avoid secondary pollution. In the current study, Ag(1%)-TiO_2_/H_3_PW_12_O_40_(10%) film was selected to conduct the recycling experiment under the optimum condition for three times, the composite film was dipped in ethanol to remove the absorbed o-CP molecules after each catalytic run, then washed by distilled water and dried at room temperature. The results showed that even after 3 times recycle, the composite film could still degrade more than 80.00% of o-CP (Fig. 21), and only 0.13% H_3_PW_12_O_40_ and 0.05% Ag dropped from the film.

**Figure 22:**
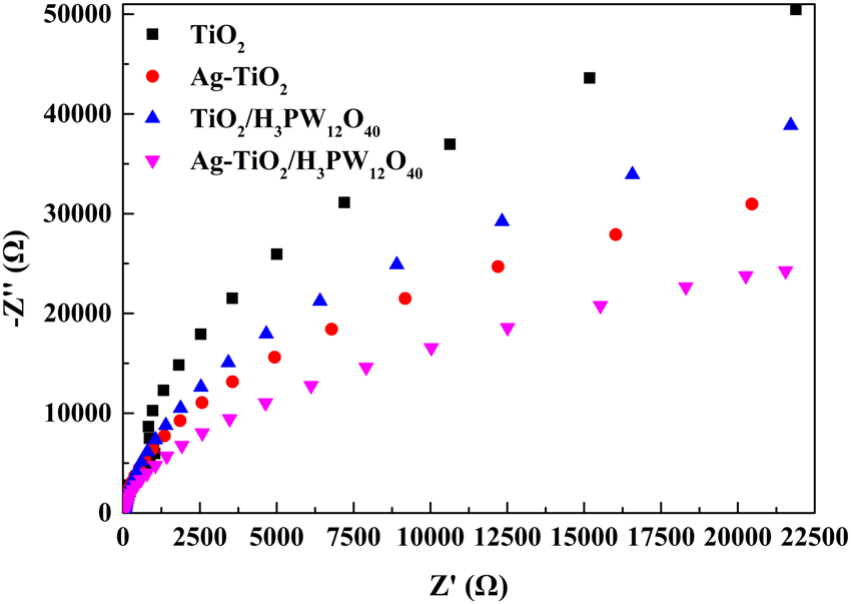
EIS Nyquist plots of TiO_2_, Ag-TiO_2_, TiO_2_/H_3_PW_12_O_4_, and Ag-TiO_2_/H_3_PW_12_O_40_ film.

The electrochemical impedance spectroscopy (EIS) of TiO_2_, Ag-TiO_2_, TiO_2_/H_3_PW_12_O_4_, and Ag-TiO_2_/H_3_PW_12_O_40_ film was implemented to quest their charge transport capability. It is well-known that the smaller arc radius is, the higher separation efficiency of electrons-holes becomes. As displayed in Fig. 22, arc radius of Ag-TiO_2_/H_3_PW_12_O_40_ was the smallest among all the films, suggesting the least resistance for charge transfer.

**Figure 23:**
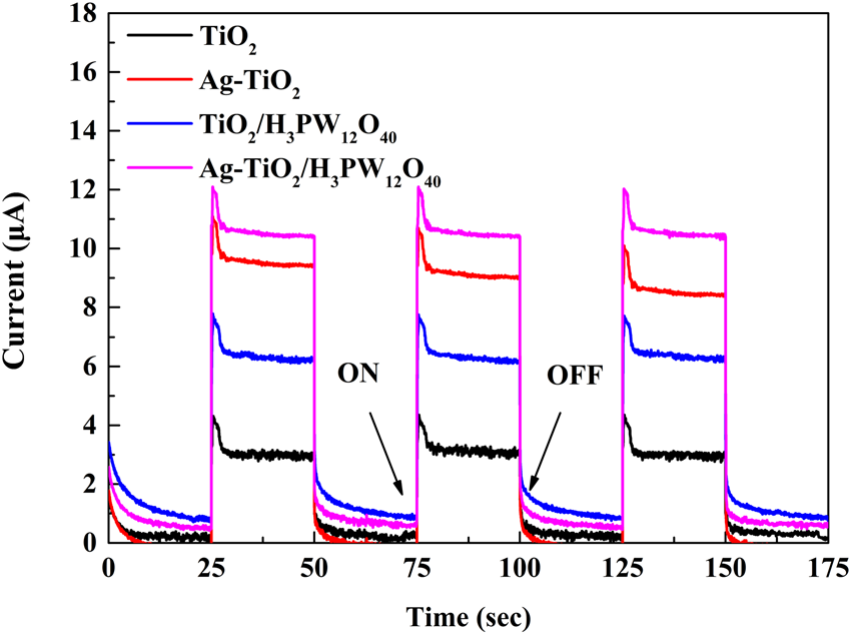
Photocurrent responses of TiO_2_, Ag-TiO_2_, TiO_2_/H_3_PW_12_O_40_ and Ag-TiO_2_/H_3_PW_12_O_40_ film electrodes.

The photocurrent-time (*I-t*) curves of TiO_2_, Ag-TiO_2_, TiO_2_/H_3_PW_12_O_4_, and Ag-TiO_2_/H_3_PW_12_O_40_ film are shown in Fig. 23 with the cycles of light-on and light-off. Distinctly, Ag-TiO_2_/H_3_PW_12_O_4_ and Ag-TiO_2_ film represented a higher photocurrent intensity during the cycles of on-off intermittent irradiation, reconfirming that the introduction of Ag into the catalyst was feasible to increase both the quantum efficiency and separation efficiency of photogenerated electron-hole pairs, which was corresponding to the results of EIS. However, the stability of Ag-TiO_2_ film was not the same as Ag-TiO_2_/H_3_PW_12_O_4_ and its photocurrent intensity decreased after every cycle of light-on and light-off, which further induced the decreasing of photocatalytic activity. This can be attributed to the fact that metal Ag can be easily oxidized after depositing on the surface of TiO_2_ particles, if the cover was absent.

Overall, an excellent photocatalytic activity, stability and reproducibility of Ag-TiO_2_/H_3_PW_12_O_4_ composite film was attained from the following approaches: (1) the excellent photocatalytic activity was attributed to a large quantity of holes and electrons produced by adsorbing simulated sunlight irradiation induced by SPR effect; (2) the enhanced quantum efficiency was owing to the strong electron-accepting capability of H_3_PW_12_O_40_ and the formation of Schottky junction via the modification with metallic Ag; (3) the excellent recyclability was due to the preferably preparation method, the self-regeneration of H_3_PW_12_O_40_ as well as loading of Ag^0^ into the TiO_2_/H_3_PW_12_O_40_ framework.

## Conclusions

An efficient plasmonic Ag-TiO_2_/H_3_PW_12_O_40_ composite film with enhanced sunlight photocatalytic activity was prepared by modified sol-gel-hydrothermal method combined with spin coating technique. It has been revealed that the composite film was an excellent photocatalytic activity towards o-CP degradation, mainly due to the extra active electrons and holes generated by SPR effect as well as Schottky junction via the modification with metallic Ag. ·OH and ·O_2_^−^ were confirmed to play an essential role in photocatalytic degradation of o-CP, and the possible o-CP photodegradation pathways were put forward according to the identified intermediate products. The mineralization testified the strong oxidation ability of Ag-TiO_2_/H_3_PW_12_O_40_ catalyst, which could decompose the contaminants into CO_2_ and H_2_O. It also showed a remarkably excellent stability and recyclability of the composite film in degrading o-CP, which may greatly limit the economic cost and secondary pollution. The studies in this work provide important information on o-CP degradation, which will promote the technical development for its removal. The plasmonic composite film could be used further for the decomposition of persistent organic pollutants with low concentration in practical water and wastewater treatment.

## Electronic supplementary material

Supplementary information
