# Supplementary material for: Design of plasmonic Ag-TiO2/H3PW12O40 composite film with enhanced sunlight photocatalytic activity towards o-chlorophenol degradation
Source: Sci Rep. 2017 Dec 11;7:17298. doi: 10.1038/s41598-017-17221-4 (PMC5725600; doi:10.1038/s41598-017-17221-4)
Supplement: Supplementary file 1 — Supplementary information [file 41598_2017_17221_MOESM1_ESM.doc]

**Design of plasmonic Ag-TiO_2_/H_3_PW_12_O_40_ composite film with enhanced sunlight photocatalytic activity towards *o*-chlorophenol degradation**

Nan Lu^a†^, Yaqi Wang^a†^, Shiqi Ning^a^, Wenjing Zhao^a^, Min Qian^b^, Ying Ma^a^, Jia Wang^a^, Lingyun Fan^a^, Jiunian Guan ^a*^, Xing Yuan^a**^

^a^ School of Environment, Northeast Normal University, Changchun 130117, P. R. China

^b^ College of Chemistry, Northeast Normal University, Changchun 130117, P. R. China

†: These authors contributed equally to this work.

*: Corresponding author. Email: guanjn461@nenu.edu.cn (J. Guan), Tel.: +86-431-89165610, Fax: +86-431-89165621.

**: Corresponding author. E-mail addresses: yuanx@nenu.edu.cn (X. Yuan), Tel.: +86-431-89165600, Fax: +86-431-89165621.

**Supporting Information:**

**Fig. S1** The adsorption of o-CP on TiO_2_, TiO_2_/H_3_PW_12_O_40_ and Ag-TiO_2_/H_3_PW_12_O_40_.

**Fig. S2** The adsorption of o-CP on Ag-TiO_2_/H_3_PW_12_O_40_ with different H_3_PW_12_O_40_ loading amount.

**Fig. S3** The adsorption of o-CP on Ag-TiO_2_/H_3_PW_12_O_40_ with different Ag loading amount.

**Fig. S4** The direct photolysis of o-CP with different initial concentration under simulated sunlight irradiation.

**Fig. S5** The adsorption of o-CP with different initial concentration on Ag-TiO_2_/H_3_PW_12_O_40_.

**Fig. S6** The adsorption of o-CP with different initial pH values on Ag-TiO_2_/H_3_PW_12_O_40_.

**Fig. S7** Direct photolysis of o-CP with different initial pH values under simulated sunlight irradiation.


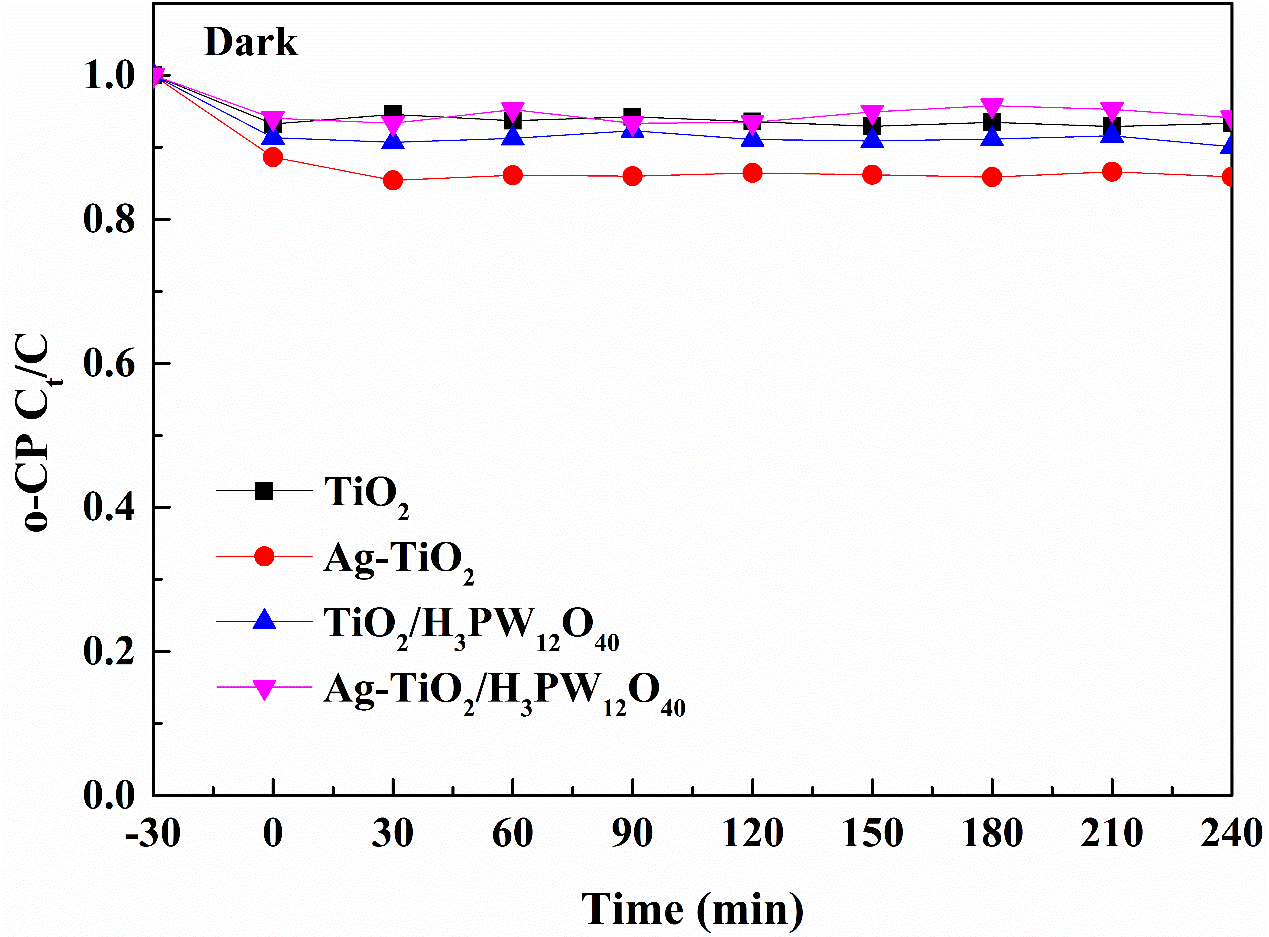


**Fig. S1** The adsorption of o-CP on TiO_2_, TiO_2_/H_3_PW_12_O_40_ and Ag-TiO_2_/H_3_PW_12_O_40_.


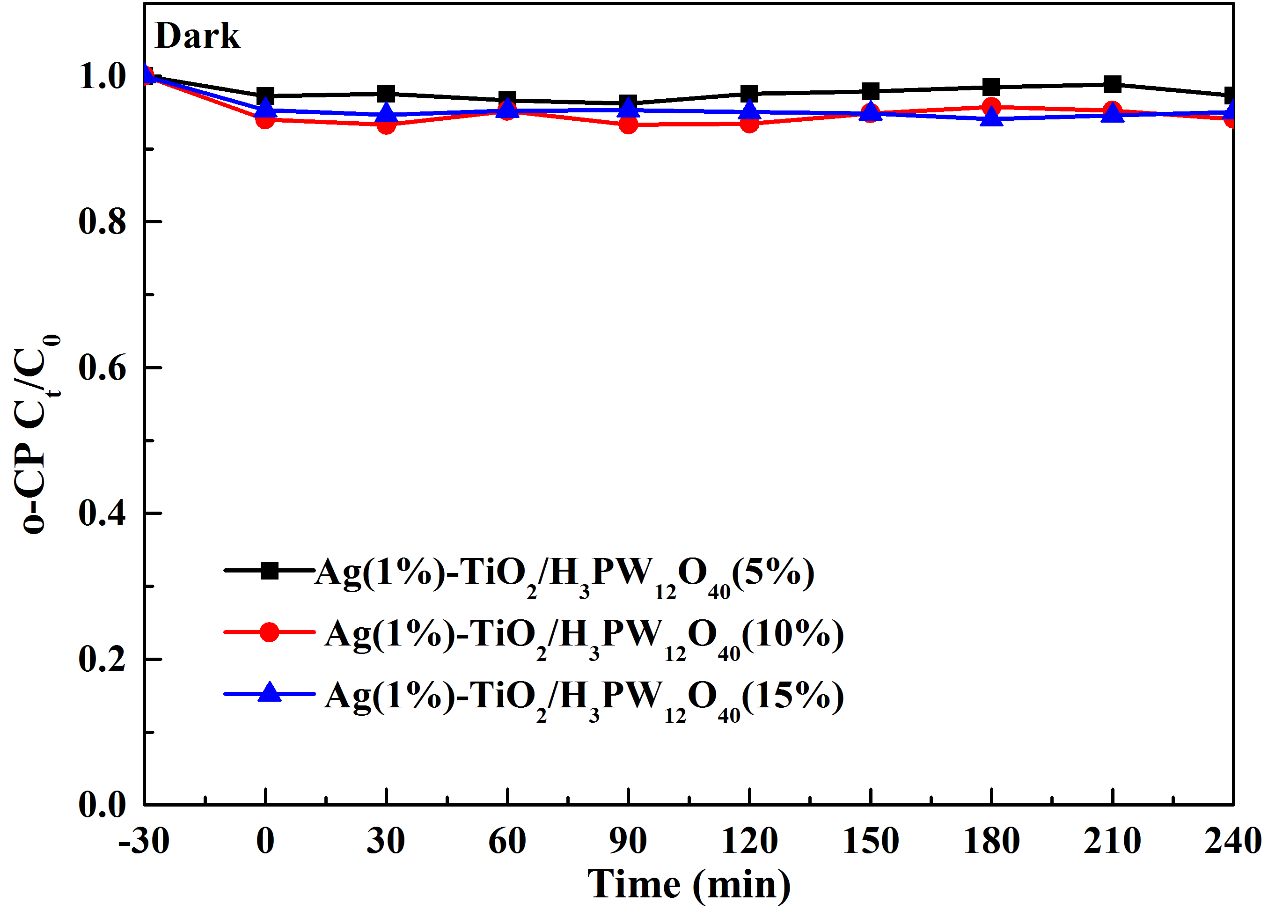


**Fig. S2** The adsorption of o-CP on Ag-TiO_2_/H_3_PW_12_O_40_ with different H_3_PW_12_O_40_ loading amount.


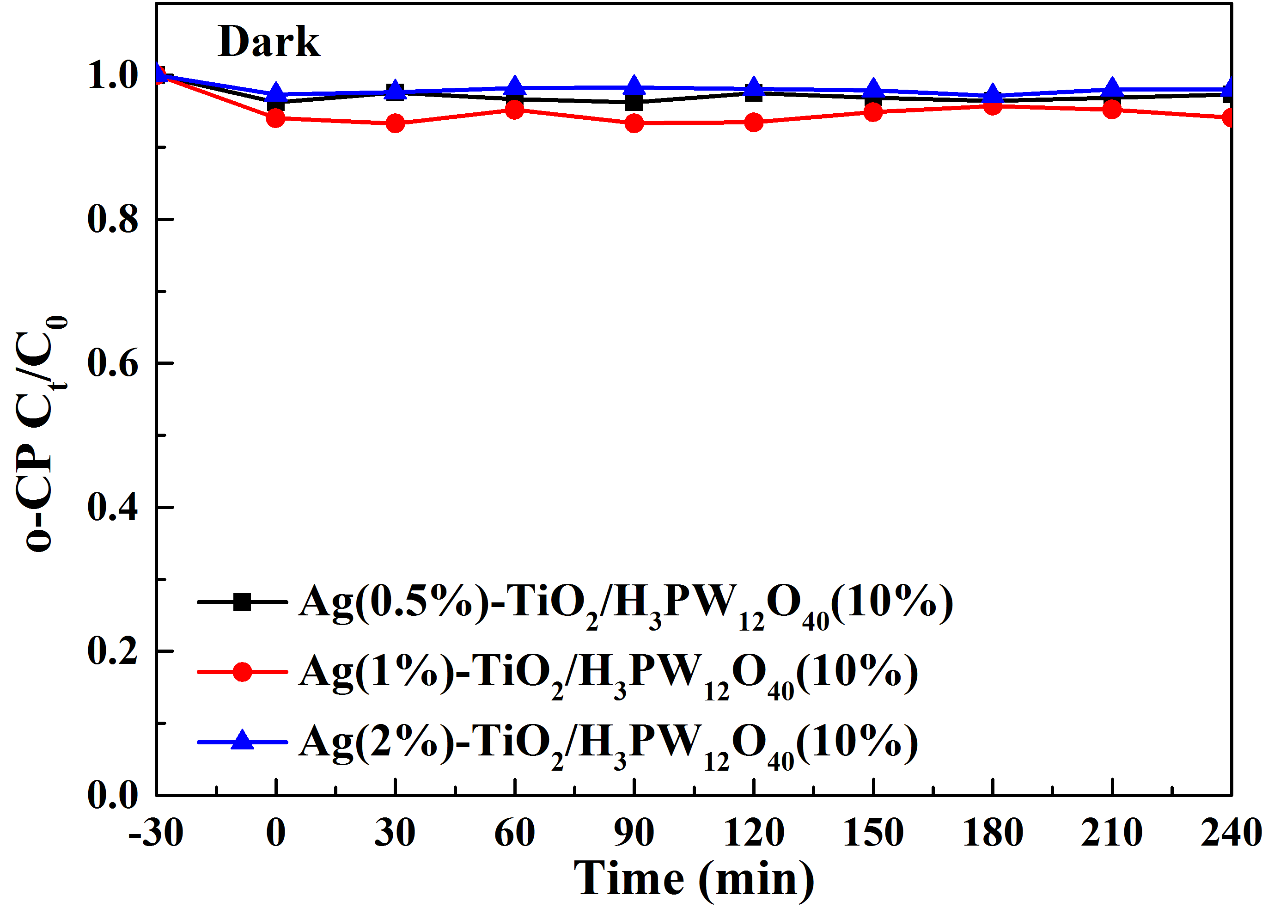


**Fig. S3** The adsorption of o-CP on Ag-TiO_2_/H_3_PW_12_O_40_ with different Ag loading amount.


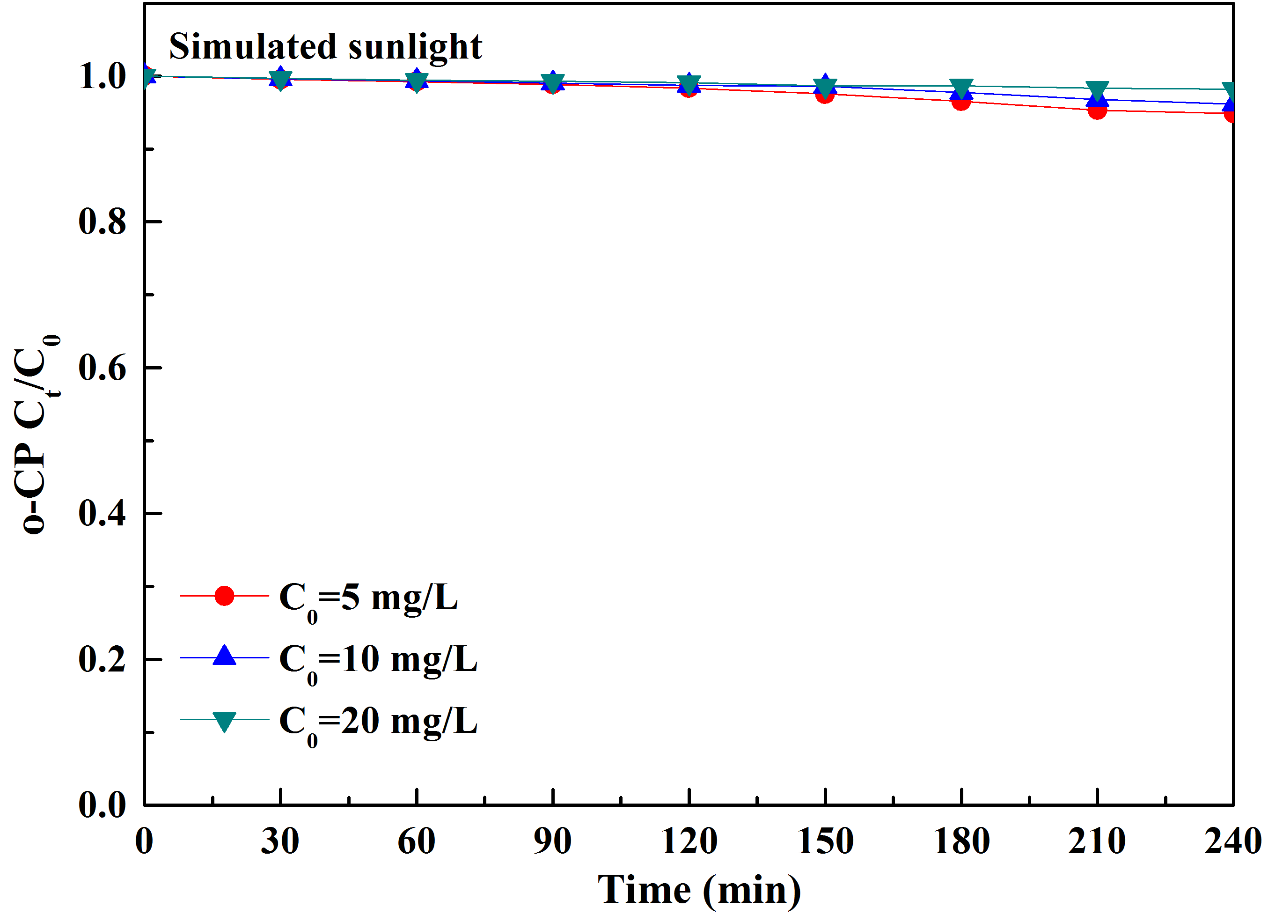


**Fig. S4** The direct photolysis of o-CP with different initial concentration under simulated sunlight irradiation.


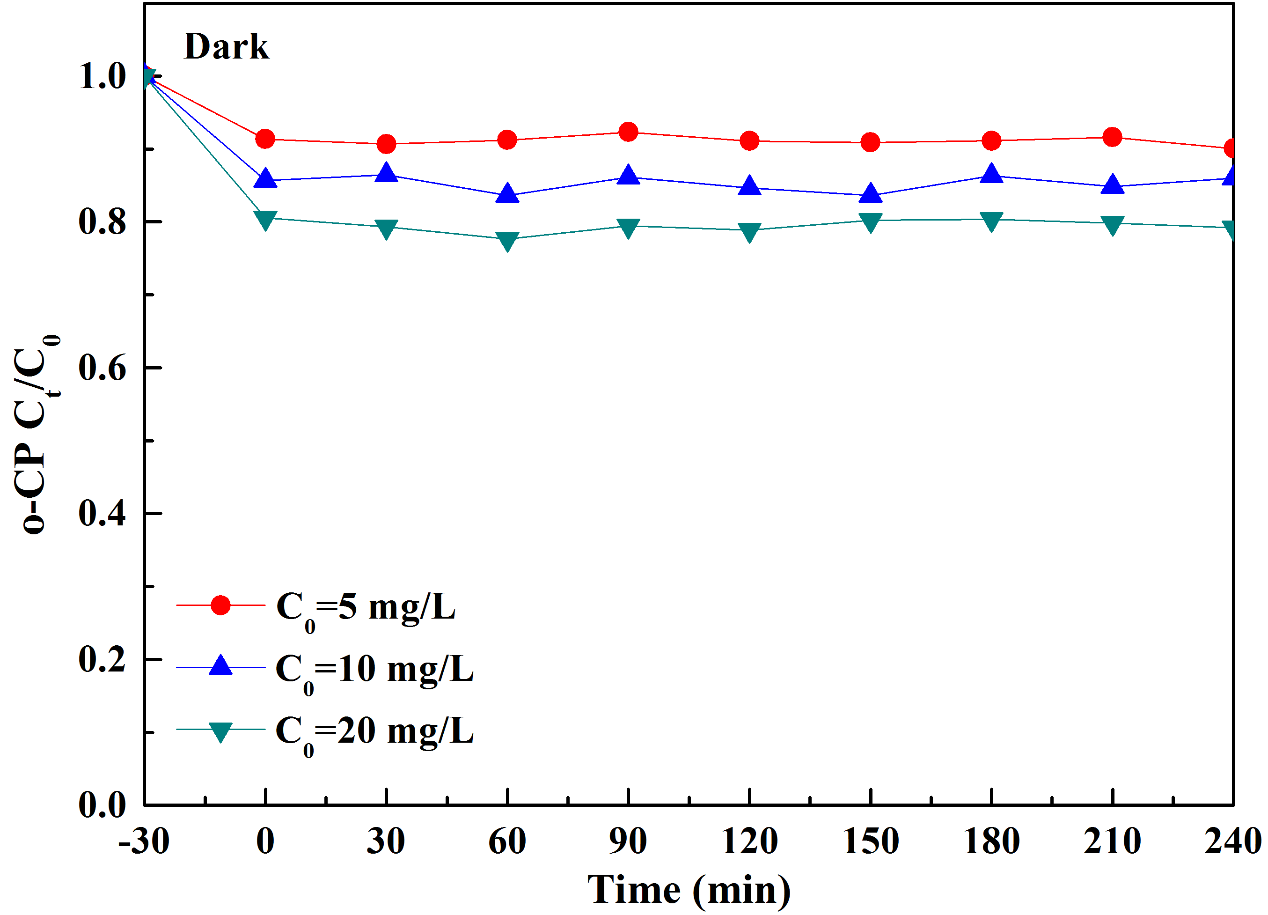


**Fig. S5** The adsorption of o-CP with different initial concentration on Ag-TiO_2_/H_3_PW_12_O_40_.


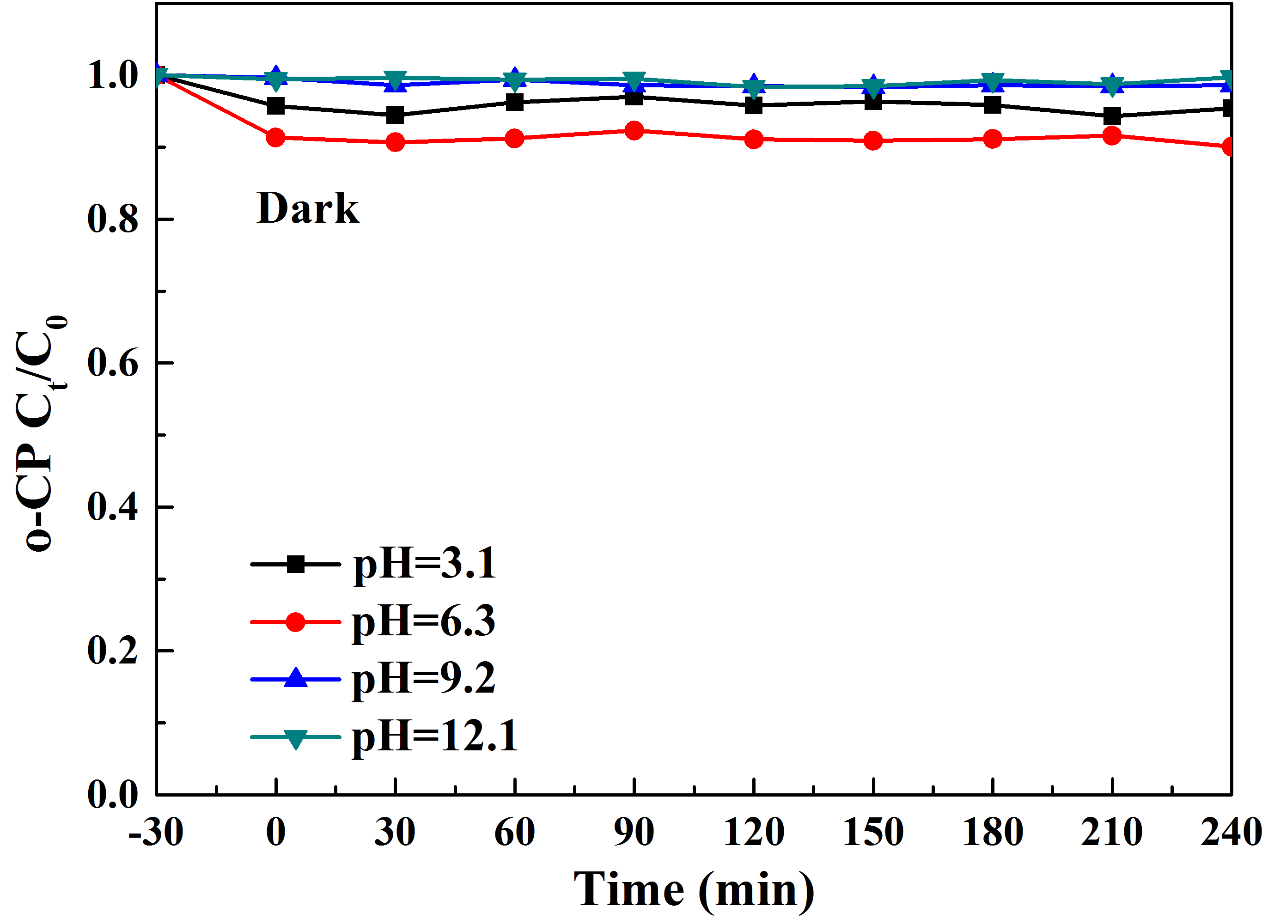


**Fig. S6** The adsorption of o-CP with different initial pH values on Ag-TiO_2_/H_3_PW_12_O_40_.


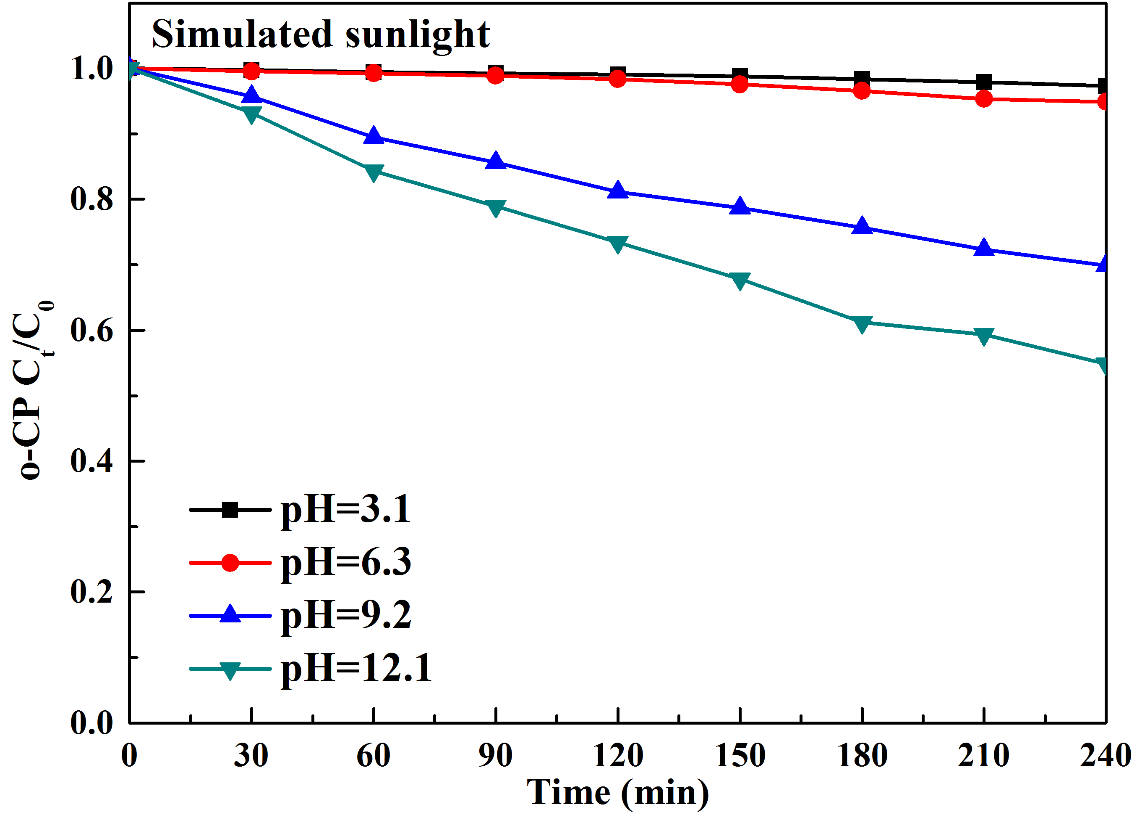


**Fig. S7** Direct photolysis of o-CP with different initial pH values under simulated sunlight irradiation.
